# Splice Type‐Specific Effects of Gαo Subunits on Cerebellar Anatomy and Synapse Formation

**DOI:** 10.1111/jnc.70512

**Published:** 2026-07-02

**Authors:** Markus Höltje, Anton Wolkowicz, Gudrun Ahnert‐Hilger

**Affiliations:** ^1^ Institut für Integrative Neuroanatomie, Charité‐Universitätsmedizin Berlin, Corporate Member of Freie Universität Berlin and Humboldt‐Universität Zu Berlin Berlin Germany; ^2^ Laboratory of Neurobiology, Max‐Planck‐Institute for Biophysical Chemistry and University of Göttingen Göttingen Germany

**Keywords:** cerebellum, Gα_o1_, Gα_o2_, VGAT, VGLUT2

## Abstract

This study investigated the effect of single Gα_o1_ and Gα_o2_, as well as double Gα_o1/2_ knockout on the cerebellar anatomy and synapse formation. The alpha subunit of the G protein Go exists in two splice variants. Knockout of certain Gαo subtypes result in strong—mainly motor—deficits in mice, and mutations in the responsible gene locus in humans can result in severe encephalopathies. We aimed to decipher the hitherto incompletely understood contribution of the individual Gα_o_ subunits to the anatomy and synapse formation of the cerebellum. Knockout of Gα_o1_ reduced the size of the cerebellum by 11%, accompanied by maximal reductions of the molecular layer thickness in the central lobule III (−30%) and molecular layer area in the uvula (−33%). Knockout of Gα_o2_ increased cerebellar size, molecular layer thickness in central lobule II (+18.6%), and area of the culmen (+37%). Combined deletion of Gα_o1_ and Gα_o2_ reduced cerebellar size by 12%, molecular layer thickness and area of the declive (by −27.3% and −23.4%, respectively). Moreover, VGLUT2‐positive climbing fiber contacts to Purkinje cells were reduced in Gα_o1_ knockout mice (on average by 40%). Similarly, VGLUT1 expression was reduced (on average by 17.3%). The knockout of Gα_o2_ promoted climbing fiber contacts (+14.3% on average, at a maximum of +52.6% in the central lobule II), VGLUT1 was less affected. Double knockout mice exhibited negative effects on VGLUT2 (−35% overall number) and VGLUT1 (−23% on average in expression levels). VGAT‐positive synaptic contacts were also diminished for Gα_o1_ and double knockout (−25% and −31% overall number, respectively) and increased for Gα_o2_ knockout (+13% on average). In line with this, negative effects on the dendritic outgrowth of Purkinje cells were observed in both Gα_o1_^−/−^ and Gα_o_^−/−^mice, while knockout of Gα_o2_ promoted dendrite outgrowth. Taken together, the two Gαo splice variants contrarily contribute to the development of the cerebellum, with Gα_o1_ representing the dominant subunit.

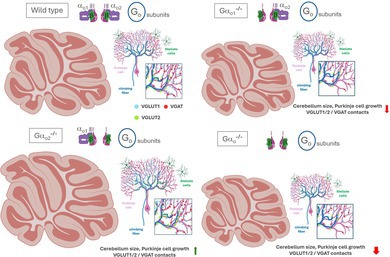

## Graphical abstract

Heterotrimeric Go alpha subunits are widely expressed in the brain to act as molecular switches. In this study we show that Gα_o1_ and Gα_o2_ affect cerebellar morphology and synapse formation in an opposite manner. While knockout of Gα_o1_ results in cerebellar hypoplasia and reduced synaptic input to Purkinje cells by VGLUT2‐positive brainstem climbing fibers, VGLUT1‐positive parallel fibers, and inhibitory inputs from GABAergic interneurons, deletion of Gα_o2_ rather promotes these parameters. The double knockout resembles the single Gα_o1_ knockout, demonstrating a dominant function of Gα_o1_

**Abbreviations**
Calb: calbindin
CENT: central lobule
CNS: central nervous system
CUL: culmen
DEC: declive
FOTU: fotulum‐tuber‐vermis
GTP: guanosine triphosphate
NOD: nodulus
PYR: Pyramis
UVU: uvula
VGAT: vesicular GABA transporter
VGLUT1: vesicular glutamate transporter 1
VGLUT2: vesicular glutamate transporter 2
WT: wild type

## 1 Introduction

Upon ligand binding of G‐protein‐coupled receptors, heterotrimeric G‐proteins exert their cellular functions via GTP binding and dissociation of the Gα subunit from the Gβγ subunits to further induce downstream signaling (Pierce et al. 2002). Since G‐proteins share a common pool of Gβγ subunits, specificity of signaling is conveyed by the 16 different Gα subunits identified so far (Wettschureck and Offermanns 2005). Among them, Gαo is predominantly expressed in the CNS and represents one of the most abundant brain proteins (Sternweis and Robishaw 1984; Solis and Katanaev 2017). The two existing splice variants Gα_o1_ and Gα_o2_ are derived from a single gene termed *GNAO1* in humans (Hsu et al. 1990). Reflecting the high abundance of Gαo proteins in the brain, failure of Gαo signaling can result in a strong phenotype. Certain mutations of the *GNAO1* gene in humans lead to severe so‐called *GNAO1* encephalopathies, mainly manifesting in epileptic episodes and movement disorders, but also intellectual impairment (Decraene et al. 2024; Ludlam et al. 2024). So far, it is largely unknown as to what extent the two isoforms Gα_o1_ and Gα_o2_ might differentially contribute to the diseases. In animal models, however, strong evidence is mounting up that the two splice products of the same gene might serve different, opposing, functions in the brain. Our group has previously shown that knockout of Gα_o1_, but not Gα_o2_, resulted in motor deficits in mice, accompanied or even caused by an imbalance in the striatal dopaminergic system (Baron et al. 2018). Opposing effects on the growth of cultured neurons were detected in a way that knockout of Gα_o1_ reduced axonal and dendritic outgrowth while depletion of Gα_o2_ resulted in stimulating effects on these parameters (Baron et al. 2013; Höltje et al. 2025). Double knockout of Gα_o1_ and Gα_o2_ had no effects on the morphology of cultured neurons. In line with this, the size of the synaptic termination fields of the hippocampal mossy fiber tract was reduced by Gα_o1_, but double knockout of both Gα_o_ isoforms largely rescued the in vivo effect (Höltje et al. 2025). Generally, Gα_o1_ knockout mice are smaller than their wild type littermates and exhibit a markedly reduced life span, while Gα_o2_ knockout mice show a slightly increased weight and a normal life span.

Given the observations of motor deficits in Gα_o1_ knockout mice and the known fact that deletion of the whole Gα_o_ protein results in an impairment of the cerebellar cortical development (Cha et al. 2019) we wanted to further elucidate the individual roles of Gα_o_ isoforms for cerebellar anatomy and synaptic connectivity. Expression of Gα_o_ is highly abundant in the molecular layer of the cerebellum and alterations in the expression profile of various Gα subunits (including also Gα_i_ and Gα_q_) have been shown to shape Purkinje cell development (Schüller et al. 2001). Besides its well‐known ubiquitous localization at the neuronal plasma membrane, tandem affinity purification methods identifying binding partners of the postsynaptic density protein of 95 kDa (PSD‐95) have also localized Gα_o1_ to the postsynaptic compartment (Fernández et al. 2009). A reduced complexity and growth of Purkinje cell dendrites together with a reduced number of VGLUT2‐positive climbing fibers contacting the Purkinje cell dendrites was already demonstrated for the double knockout (Cha et al. 2019).

Therefore, to address the differential role of Gα_o1_ and Gα_o2_ for the functional architecture of the cerebellum as a pivotal brain area to shape motor functions, we determined their effects on the anatomy of the cerebellar cortex with respect to the individual folia in paramedian sagittal sections of the cerebellar vermis by using single Gα_o1_, Gα_o2_, and double Gα_o1/2_ knockouts. Furthermore, we investigated the distribution and size of excitatory olivary climbing fiber synapses within the molecular layer of the individual folia of the cerebellar cortex in the various mutants. Besides this, we investigated effects on the VGLUT1‐positive synapses formed by the parallel fibers to the Purkinje cells. Additionally, the same parameters were studied for inhibitory connections within the molecular layer, mainly provided by GABAergic interneurons such as stellate and basket cells to Purkinje cells. Last but not least, knockout effects on Purkinje cell morphology were studied.

## 2 Materials and Methods

### 2.1 Animals

Gα_o1_^−/−^ (NIE/Birnb Go1alpha 129SV/C57BJ/6), Gα_o2_^−/−^ (NIE/Birnb Go2alpha 129SV/C57BJ/6), and Gα_o1/2_^−/−^ (NIE/Birnb Goalpha 129SV/C57BJ/6) splice variant‐specific mutant mice were obtained from the lab of Lutz Birnbaumer, bred in the local animal facility and genotyped as described (Jiang et al. 1998; Dhingra et al. 2002). Mutant and wild type (WT) littermates were obtained by interbreeding of heterozygous parents. Institutional approval (approval number T0119/11) was obtained for sacrificing the animals used in this study. Adult animals were kept with 3–4 cage companions in a space of 360 cm^2^, enriched with a hiding place, nest‐building material and ad libitum access to water and food. Pups were kept with their mother before sacrificing.

### 2.2 Cerebellar Cryosections and Immunofluorescence

20 μm sagittal cryosections from 3 to 4 adult (3–6 month of age) perfusion‐fixed animals of either sex per genotype were analyzed. Prior to perfusion, mice were anesthetized by subcutaneous injection of 10–12 mg/100 g ketamine and 1–1.5 mg/100 g xylazine. Following anesthesia, intracardiac perfusion into the left ventricle via a 26G cannula with Ringer's infusion solution as a pre‐flush solution for 3 min was done. Fixation was achieved using 4% formalin in phosphate buffer for 20 min. Subsequently, brains were removed for cryoprotection and examination. In total, 27 adult mice (9 per specific knockout and littermate wild type) had to be sacrificed. Per brain, 6–8 sections from wild type/knockout animals were typically selected from equivalent regions of the cerebellar vermis using anatomical landmarks, including size and shape of the individual folia. The sections were washed with PBS and incubated in blocking solution (10% NGS in PBS; 0.3% Triton‐X‐100) for 30 min at room temperature. The primary antibodies were diluted in primary antibody‐solution (10% NGS in PBS; 0.3% Triton‐X‐100; 0.1% NaN_3_), added to the brain sections and incubated for 24 h at 4°C. After incubating the sections, they were washed with PBS and subsequently incubated with secondary antibodies in secondary antibody‐solution (5% NGS in PBS; 0.1% Triton‐X‐100) for 1 h at room temperature in the dark. The sections were washed with PBS, stained with DAPI and mounted to the slides.

### 2.3 Antibodies

Synaptic connections of brain stem climbing fibers to Purkinje cell dendrites were stained by a recombinant Guinea pig antibody directed against the vesicular glutamate transporter 2 obtained from Synaptic Systems (VGLUT2, cat. no. 135418, Göttingen, Germany). Imaging of vesicular glutamate transporter 1 (VGLUT1) was done using a polyclonal Guinea pig antibody (cat. no. 135304, Synaptic Systems). For visualization of the vesicular GABA transporter, a recombinant Guinea pig antibody was applied (Synaptic Systems, VGAT, cat. no. 131308). Morphology of Purkinje cells was visualized by a polyclonal antiserum against Calbindin purchased from Swant AG (cat. no. CB38a, Burgdorf, Switzerland). Parvalbumin‐positive cell counts were performed using a rabbit polyclonal antiserum (cat. no. PV‐28, Swant AG). A monoclonal antibody against both splice variants of G*α*_o_ (G*α*_o1_ and G*α*_o2_, clone 101.1) was previously described (Winter et al. 2005). A polyclonal antibody preferentially recognizing Gα_o1_ was from Santa Cruz (cat. no. 13532, Santa Cruz, CA, USA). Monoclonal antibodies specifically recognizing Gα_o2_ were from Synaptic Systems (cat. no. 271011, clone 101.4, Western blot) and 201.2 (kind gift from Karsten Spicher, see Spicher et al. 1992, immunofluorescence). A monoclonal anti Synaptobrevin antibody was from Synaptic Systems (cat. no. 104211). Secondary antibodies were Alexa 594 goat anti rabbit and Alexa 488 goat anti Guinea pig (cat. nos. A‐11037 and 11 073, Thermo Fisher, Waltham, MA, USA). An Alexa 594 goat anti mouse antibody was also from Thermo Fisher (cat. no. A‐11005). Red fluorescence is displayed in magenta in all figures. For Western blotting, peroxidase‐conjugated anti‐mouse and anti‐rabbit IgG were obtained from Vector Laboratories (cat. nos. PI‐2000 and PI‐1000; Newark, CA, USA).

### 2.4 Image Acquisition for Synaptic Analysis

Microscopic images were obtained using either a confocal Leica SL microscope at 40× magnification (VGLUT1) or a Leica DMLB epifluorescence microscope (VGLUT2 and VGAT) at 20× magnification. The images were captured and stored in an 8‐bit grayscale format to maintain consistency and facilitate accurate analysis.

#### 2.4.1 Preprocessing

The acquired images were processed using open source software ImageJ. To enhance visibility and isolate synapses in the molecular layer, a threshold was applied. This threshold was carefully validated by comparing the processed images with the original microscopic images to ensure optimal settings for precise synapse identification. The same settings were applied consistently across all images of a single animal to ensure comparability in the results.

#### 2.4.2 Region of Interest (ROI) Selection

For each section, a 200 μm (VGLUT2, VGAT) or 150 μm (VGLUT1) wide region was manually drawn, covering the total height of the molecular layer (see also Figures 2D,G,J and 4F–H). This ensured that only the relevant region was analyzed, improving accuracy in detecting synaptic structures.

#### 2.4.3 Automated Analysis in ImageJ

A custom macro was developed in ImageJ to automate the analysis process. It iterated through all sections in the stack, activated the corresponding ROI for each section, and executed the “Analyze Particles” function within the defined ROI to extract the total count and area of detected particles. The “Analyze Particles” settings were configured with a size range of 0 to infinity μm^2^, a circularity of 0.00–1.00, pixel units enabled, and the summarize function activated while displaying no additional outputs. The summary results generated by ImageJ were exported for further statistical analysis. The data was structured and organized in Excel to facilitate statistical comparisons (see below).

#### 2.4.4 Quantification of VGLUT1 Expression

Given the very high level and density of VGLUT1 expression in the molecular layer, systematic analysis of protein expression was based on brightness analysis in addition to the above described procedure, that was also done in the case of VGLUT1 for one selected lobule per genotype. Cerebella were imaged at 10× magnification using epifluorescence and the average brightness values of the VGLUT1 antibody signal within the total molecular layer of the individual folia set as a ROI were analyzed using the histogram tool of Adobe Photoshop 2021 Version 22.0. Regions compromising correct brightness values like overlapping tissue sections or increased brightness areas at the surface of the molecular layer were excluded from the analysis.

### 2.5 Cell Counting of Parvalbumin‐Positive Interneurons in the Molecular Layer

Parvalbumin‐positive cells were counted in the molecular layer using whole fields of view of the whole layer at 350 μm width taken at 40× magnification. Dapi staining was used to verify that cell body shaped Parvalbumin signals indeed represented neuronal somata of stellate and basket cells.

### 2.6 Quantification of Gαo Subunit Expression

Analysis was performed as described for VGLUT1 analysis based on brightness values.

### 2.7 Purkinje Cell Morphology

To detect knockout effects on Purkinje cell morphology, confocal imaging of Calbindin stainings was applied to determine the thickness of primary dendrites at a maximal distance of 50 μm from the soma at a section free of branch points using the Image J straight line tool.

To address overall dendritic complexity, rectangular regions of interest (ROI) at 180 × 00 μm within the molecular layer were analyzed. Images were acquired at 40× magnification, thresholded, and quantified by Image J, again using the “Analyze Particles” function to obtain the total area and proportion of the Calbindin signal within the ROI.

### 2.8 Area Measurements

The total area of the selected region of interest in the cerebellum was determined using ImageJ on microscopic images taken at 5× magnification from DAPI‐stained sections. For each folium, the area of the molecular layer and the granular layer was manually outlined using the polygon tool in ImageJ. Perimeter and area values were extracted from ImageJ for further calculations. In addition, the thickness of the molecular layer and granule cell layer was determined at constant defined positions of the individual folia. The extracted area values for each section were compiled in Excel for further analysis.

### 2.9 Western Blotting

For immunoblotting, whole brains from adult wild type, Gα_o1_^−/−^, Gα_o2_^−/−^, and Gα_o_^−/−^ mice sacrificed by cervical dislocation were homogenized (tissue was processed in PBS using a glass‐Teflon homogenizer applying 10 strokes at 900 rpm with protease inhibitors added). Homogenates were spun down at 1500 × *g* for 10 min, and the resulting supernatant devoid of cell nuclei was diluted in Laemmli buffer and submitted to SDS‐PAGE. To this end, samples were dissolved in Laemmli buffer, heated for 5 min at 95°C and transferred to Nitrocellulose membranes by semi‐dry blotting using 10% SDS gels. The membranes containing the transferred proteins (10 μg total protein per lane, determined by the bicinchoninic assay method) were blocked with 5% dry milk, 0.1% Tween‐20 in 20 mM Tris for 1 h at RT, followed by incubation with the respective primary antibodies overnight; secondary antibodies were applied for 2 h at room temperature. Washing between steps was carried out with 20 mM Tris buffer. Visualization was performed by Enhanced Chemiluminescence; imaging was done using the MultiImage Light Cabinet (Alpha Innotech Corporation, San Leandro, CA, USA) and Adobe Photoshop.

### 2.10 Statistical Analyses

Consistently, image acquisition and analysis were performed by two different investigators. Assessment of the normality of data was verified by the Shapiro–Wilk test for all data prior to parametric testing (Graph Pad Prism 8.0). For all parameters looked at, *p* values were greater than the chosen alpha level of 0.05. Statistical significance for knockout effects on cerebellar morphology and synaptic connectivity was determined by a two‐tailed, unpaired Student's *t*‐test on the basis of a significance level of *p* < 0.05 (MS Excel 2019). No test for outliers was conducted. For predetermination of sample size, experiences from former publications of our group dealing with the effects of Go proteins were used (Baron et al. 2013, 2018; Brunk et al. 2008). In bar chart graphs, mean values from pooled animals are shown. If the number of individual data points did not interfere with legibility of the graph, individual values are also given. A full statistical report is given in the Supporting Information.

### 2.11 Ethics Statement

Animal housing as well as all experiments using animal‐derived tissue/cells were performed in accordance with institutional (Charité–Universitätsmedizin Berlin Germany), local (LaGeSo, approval numbers O 0062‐07 and T0119‐11), and national guidelines (German Animal Welfare Act).

## 3 Results

### 3.1 Expression and Effects of Gα_o_ Subunit Knockouts on Overall Cerebellar Anatomy and Cortex Size of Individual Folia

**FIGURE 1:**
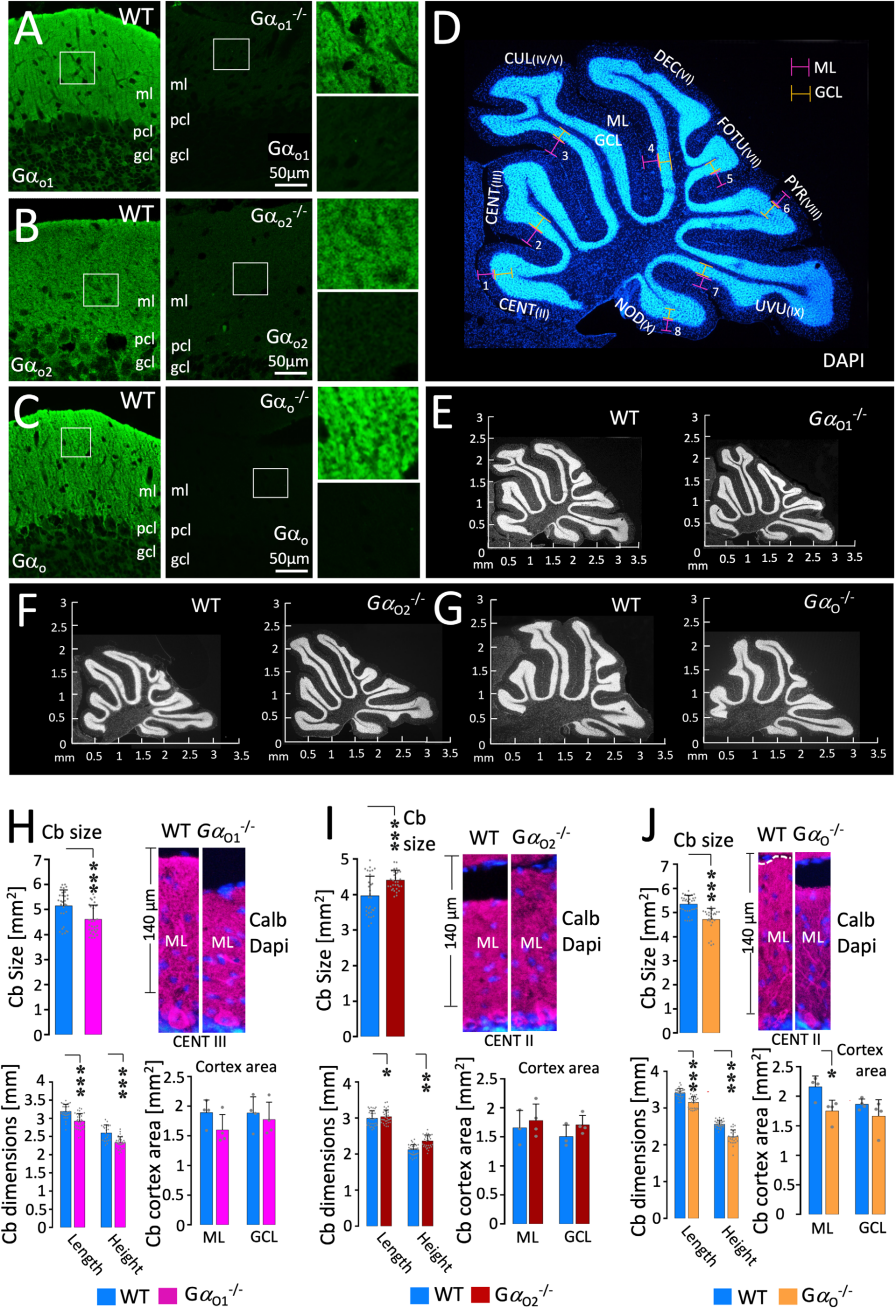
Gα_o1_ and Gα_o2_ differentially affect the cerebellar size. (A) Immunohistochemical analysis of Gα_o1_ expression in the cerebellum of wild type and Gα_o1_^−/−^ mice. Midsagittal cerebellum sections of adult mice of either strain were stained by an antibody preferentially recognizing Gα_o1_. In the wild type, Gα_o1_ showed a very strong reaction predominantly in the molecular layer. Immunostaining against Gα_o1_ was absent in the knockout. gcl, granule cell layer; ml, molecular layer; pcl, Purkinje cell layer. (B) Analysis of Gα_o2_ expression. Midsagittal cerebellum sections of adult mice were stained by an antibody recognizing Gα_o2_. In the wild type, Gα_o2_ showed a strong reaction predominantly in the molecular layer. Immunostaining against Gα_o2_ was absent in the knockout. (C) Analysis of Gα_o1/2_ (Gα_o_) expression. Midsagittal cerebellum sections of adult mice of either strain were stained by an antibody recognizing both Gα_o1_ and Gα_o2_. In the wild type, Gα_o_ showed a very strong reaction predominantly in the molecular layer. Immunostaining against Gα_o_ was absent in the knockout. (D) An exemplary midsagittal DAPI‐stained section of wild type cerebellum with the individual folia labeled. Cranial orientation is to the left. CENT, central lobule; CUL, culmen; DEC, declive; FOTU, fotulum‐tuber‐vermis; NOD, nodulus; PYR, pyramis; UVU, uvula (lobule numbers are given in latin). (E) Comparison of wild type (as shown in D) and Gα_o1_^−/−^ cerebellum size. (F) Comparison of wild type and Gα_o2_^−/−^ cerebellum size. (G) Comparison of wild type and Gα_o_^−/−^ cerebellum size. (H) Quantification of overall cerebellar size (given in mm^2^, upper left panel) along with the cranio‐caudal (length) and ventro‐dorsal (height) extension (lower left panel). In addition, the cortical area was calculated for the molecular and granule cell layer (lower right panel). See effects exemplified for CENTIII by Calbindin staining. (I) The same analysis was performed for Gα_o2_. See effects exemplified for CENTII by Calbindin staining, (J) Analysis for Gα_o_. See effects exemplified for CENTII by Calbindin staining. Bars show means ± SD and individual values, *N* = 4 animals each WT and knockout (except for 3 animals in Gα_o2_ WT total ML and GCL area analysis), 6–8 sections per animal and genotype. *p ≤ 0.05; **p ≤ 0.01; ***p ≤ 0.001.

First, we compared various size parameters, including length, height, cortical, and overall area of the cerebellum, complimented by an analysis of the thickness and area size of the molecular and granule cell layer with respect to the individual folia as observed in midsagittal sections of the cerebellar vermis in the respective mutants. Previous work by our group had shown that both splice isoforms can exhibit opposing effects on neuronal outgrowth and synapse formation in the hippocampus (Höltje et al. 2025). Initially, expression of Gα_o1_ in the wild type cerebellum and absence of protein in the knockout was verified by immunofluorescence. An antibody preferentially recognizing Gα_o1_ (Höltje et al. 2025) detected a strong expression in the wild type molecular layer, other areas showed lower expression levels (Figure 1A). Homozygous knockout of Gα_o1_ resulted in the complete loss of the immune signal. Pups were bred as littermates from heterozygous parents and genotyping was performed for all mice used in the analysis to confirm correct knockout and is exemplarily shown (Figure S1A). In addition, deletion of Gα_o1_ was confirmed by Western blotting. Using brain homogenates from adult wild type and Gα_o1_^−/−^ mice, specific deletion of Gα_o1_ was demonstrated (Figure S1B). Next, we investigated the expression of Gα_o2_ in the cerebellar cortex. Expression of Gα_o2_ in the wild type cerebellum and absence of protein in the knockout was also verified by immunofluorescence. An antibody recognizing Gα_o2_ detected a strong expression in the wild type molecular layer (Figure 1B). Homozygous knockout of Gα_o2_ resulted in the loss of the immune signal. Again, genotyping was performed for all littermate mice used in the analysis to confirm correct knockout of Gα_o2_ and is shown exemplarily (Figure S1A). Deletion of Gα_o2_ was also confirmed on protein level in the same way as demonstrated for Gα_o1_ (Figure S1B). Furthermore, we also sought to unravel the effects of the combined double knockout of both splice isoforms. Following verification of double Gα_o1_ and Gα_o2_ knockout (referred to as Gα_o_^−/−^) by immunohistochemistry using an antibody recognizing both isoforms (Figure 1C), genotyping (Figure S1A), as well as Western blotting of wild type and knockout brain homogenates (Figure S1B), we compared the cerebellar size parameters in wild type and the respective knockout littermates.

To this end, cerebellar wild type and knockout midsagittal sections were morphometrically analyzed based on combined Calbindin and Dapi stainings. Sections display the typical folia (see Figure 1D for morphological representation of individual folia). Calbindin was used to stain the complete Purkinje cell morphology with their characteristic dendritic arborizations in the molecular layer. Deletion of Gα_o1_ resulted in moderate changes in the overall cerebellar size (−11% total area in the knockout) reflected by similar reductions in cerebellar length and height (both reduced by 8.5% in Gα_o1_^−/−^ mice, Figure 1E,H). When analyzing the total area of the molecular and granule cell layers, however, the effects that emerged for the two cortical layers did not reach statistical significance. In contrast to the situation in Gα_o1_ knockout mice, deletion of Gα_o2_ resulted in a low to moderate increase in the overall cerebellar size (+10% total area, +1.3% length and +11.3% height, Figure 1F,I). When analyzing the total area of the molecular and granule cell layers, a tendency to higher values in the knockout was observed that did not reach statistical significance. Deletion of Gα_o_, representing the double knockout, resulted in moderate changes in the overall cerebellar size (−12% total area in the knockout) reflected by reductions in cerebellar length (−7.6%) and height (−15.3%, Figure 1G,J). When analyzing the total area of the molecular and granule cell layers, it turned out that the molecular layer was reduced by 19% in the knockout, whereas the granule cell layer effects did not reach statistical significance.

We then tested whether the individual folia exhibited differential effects as a result of the knockout. We therefore measured the thickness of the two layers at defined identical regions (see Figure 1D) in wild type and knockout animals together with the cortical area of the individual folia. It turned out that the anteriorly oriented folia central lobule II and III, as well as the culmen were generally more affected by the Gα_o1_ knockout in a reduction of thickness of the molecular layer than the other folia, being most pronounced in the central lobule III (−30% reduction in Gα_o1_^−/−^ mice, Figure S2A). Accordingly, measurements of the area covered by the molecular layer of the individual folia were performed and showed reductions in six out of eight folia including the uvula exhibiting the strongest reduction of 33% by the Gα_o1_ knockout (Figure S2B). Analysis of the granule cell layer showed no effects for the individual folia except for a small reduction in the thickness of the declive (Figure S2C). In line with this, only very low to moderate reductions in the granule cell layer area were observed in three out of the eight folia analyzed (Figure S2D). As conducted for Gα_o1_ knockout animals, we tested whether the individual folia exhibited differential effects following deletion of Gα_o2_ and measured the thickness of the molecular and granule cell layer at defined regions in wild type and knockout animals (Figure S2E,G). Supporting the findings on the overall cerebellar size, seven out of eight folia were found to be increased in their molecular layer thickness (max. +18.6% in central lobule II). Measurements of the area covered by the molecular layer of the individual folia were again performed and revealed increased values in five out of eight folia of Gα_o2_^−/−^ mice being most pronounced in the culmen (+37%, Figure S2F). Granule cell layer thickness and area were increased in five or four folia, respectively (Figure S2G,H). The detailed folia‐specific analysis of the double Gα_o_ knockout revealed a reduction of the molecular layer thickness in all folia but the uvula (with a maximum effect of −27.3% in central lobule II, Figure S2I), accompanied by reductions in the area of six folia (maximum reduction of −23.4% in the declive, Figure S2J). Reduction in the thickness and area covered by the granule cell layer were less pronounced than in the molecular layer, with four folia exhibiting a reduced thickness (mainly the anteriorly located folia at a maximal reduction of −14.5% in den central lobule III) and 3 folia with a smaller area (max. −23.3% in the declive) as shown (Figure S2K,L).

Taken together, deletion of Gα_o1_ resulted in moderate reducing effects on the overall cerebellar size by differentially affecting the cortex size parameters of individual folia towards reduced values, whereas Gα_o2_ had opposite, positive effects on many of the same parameters. The combined knockout of both isoforms created a phenotype similar to the situation in single Gα_o1_‐depleted animals, indicating an overriding importance of Gα_o1_ over Gα_o2_ regarding the growth of the cerebellum.

### 3.2 Effects of Gα_o_ Splice Isoform Knockouts on Excitatory Synaptic Contacts in the Cerebellar Cortex

**FIGURE 2:**
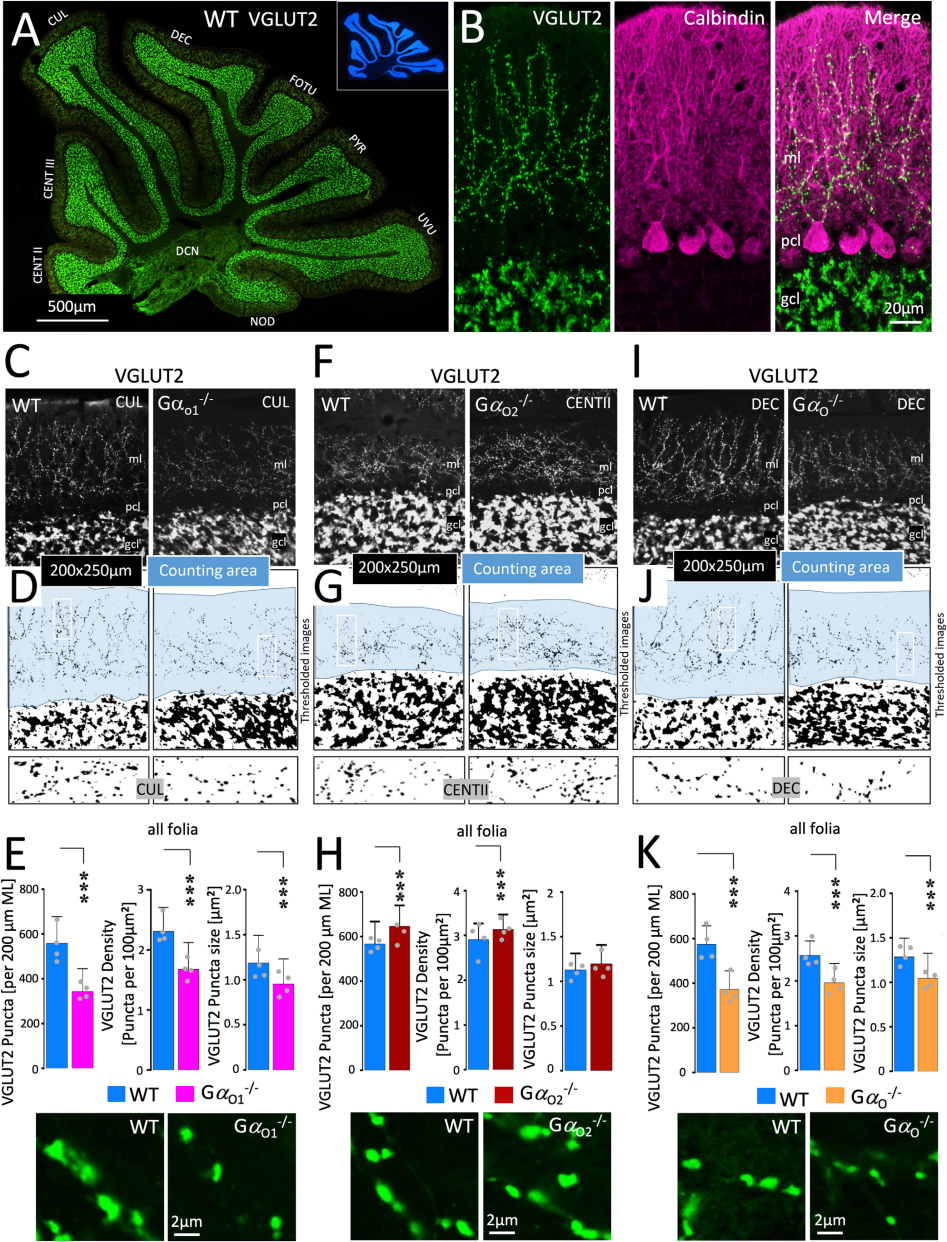
Gα_o1_ and Gα_o2_ differentially affect the number of VGLUT2 contacts within the cerebellar cortex. (A) Immunohistochemical analysis of the vesicular glutamate transporter 2 (VGLUT2) expression in the adult wild type cerebellum as shown in a midsagittal section. A strong immune signal was detected in the cerebellar glomeruli of the granule cell layer (gcl), together with a distinct punctuate pattern in the molecular layer (ml) and the deep cerebellar nuclei (DCN, see inset for Dapi staining). (B) Double staining for VGLUT2 and Calbindin to visualize Purkinje cell dendrite morphology. In the molecular layer, the VGLUT2 transporter signal characteristically marks synaptic climbing fiber contacts to the proximal to mid‐segmental Purkinje cell dendrites. (C) Regions of the molecular layer depicting VGLUT2 signals in the culmen of wild type and Gα_o1_^−/−^ mice used to quantify the VGLUT2 puncta. (D) Exemplary thresholded images of the regions shown in (C) used for analysis in the respective regions of interest (counting area, shown in transparent blue, see insets for magnification). (E) Quantification of the absolute number of VGLUT2 puncta in the counting area (left panel), the density (middle panel), and size (right panel). See higher magnification confocal imaging for details of VGLUT2 signals. (F) Analysis of the effects of Gα_o2_ knockout on the VGLUT2 staining pattern in the molecular layer of the cerebellum. Exemplary immunohistochemical visualization of VGLUT2 contacts in the central lobule II of wild type and Gα_o2_^−/−^ knockout mice. (G) Exemplary thresholded images of the regions used for quantification within the counting area (transparent blue). (H) Quantification of the absolute number of VGLUT2 puncta in the counting area (left panel), the density (middle panel), and size (right panel). See higher magnification confocal imaging for details of VGLUT2 signals. (I) Analysis of the effects of Gα_o1/2_ (Gα_o_) double knockout on the VGLUT2 staining pattern in the molecular layer of the cerebellum. Exemplary immunohistochemical visualization of VGLUT2 contacts in the declive of wild type and Gα_o_^−/−^ knockout mice. (J) Exemplary thresholded images of the above depicted regions used for quantification within the counting area (transparent blue). (K) Quantification of the absolute number of VGLUT2 puncta in the counting area (left panel), the density (middle panel), and size (right panel). See higher magnification confocal imaging for details of VGLUT2 signals. Bars show means ± SD (individual animal means are depicted), *N* = 4 animals each WT and knockout, 6–8 sections per animal and genotype. ***p ≤ 0.001.

Following the investigation of G_o_ alpha‐subunit knockout effects on cerebellar morphology we wanted to further elucidate whether the respective depletions also caused alterations in synapse establishment within the cerebellar cortex. We decided to focus on the molecular layer since it displays a very distinctive synaptic pattern regarding VGLUT2‐positive climbing fiber contacts to Purkinje cell dendrites in either a 1:1 fashion (one Purkinje cell is contacted by only one climbing fiber) or innervation by multiple climbing fibers of Purkinje cells with multiple primary dendrites (Busch and Hansel 2023), and its important inhibitory network shaping the Purkinje cell output from the cerebellar cortex to other brain regions. In addition, the extensive VGLUT1‐positive excitatory input to Purkinje cell dendrites by the granule cell parallel fibers was investigated. To address these issues, we first determined the distribution pattern of VGLUT2‐positive contacts in the individual folia of the cerebellum. By immunostaining against the vesicular glutamate transporter 2, a typical staining pattern emerges that shows a very intense staining of the synaptic structures representing a subset of the cerebellar glomeruli of the granule cell layer accompanied by a characteristic punctuate distribution pattern marking the more proximal dendritic arbor of the Purkinje cells (Figure 2A,B). We then quantified the number of VGLUT2‐positive puncta within a region of interest (ROI) of 200 μm width directly superficial to the Purkinje cell somata up to the surface of the molecular layer. Additionally, the density and puncta size were evaluated. The comparative analysis between wild type and Gα_o1_ knockout animals of the total molecular layer including all folia indicated a marked reduction of VGLUT2 puncta within the ROI by −40% in the knockout (Figure 2C–E). The observed effects were mainly due to a reduced density (calculated to −27.4%). Moreover, the size of the VGLUT2 puncta was reduced by 20% in the Gα_o1_ knockout. In analogy, we conducted the same analysis for the outcome of a Gα_o2_ depletion on VGLUT2 contacts. The evaluation of wild type and Gα_o2_ knockout animals of the total molecular layer including all folia indicated a moderately increased number of VGLUT2 puncta within the ROI by +14.3% in the knockout (Figure 2F–H). The effect was partially caused by an increased density in the knockout (+7%). The size of the VGLUT2 puncta was not significantly altered. As observed for the size of various cerebellar structures, glutamatergic contacts to Purkinje cell dendrites by VGLUT2‐positive climbing fibers are affected in an opposing manner by deleting either Gα_o1_ or Gα_o2_. To determine the depletion effects of the double knockout we again investigated the expression pattern of VGLUT2 in Gα_o_^−/−^ mice in comparison to the wild type. Knockout of Gα_o1_ and Gα_o2_ resulted in a decreased number of VGLUT2 puncta per ROI (−35%), a decreased density (−24%), as well as a reduced puncta size (−18.6%, Figure 2I–K) averaged for the molecular layer of all folia.

**FIGURE 3:**
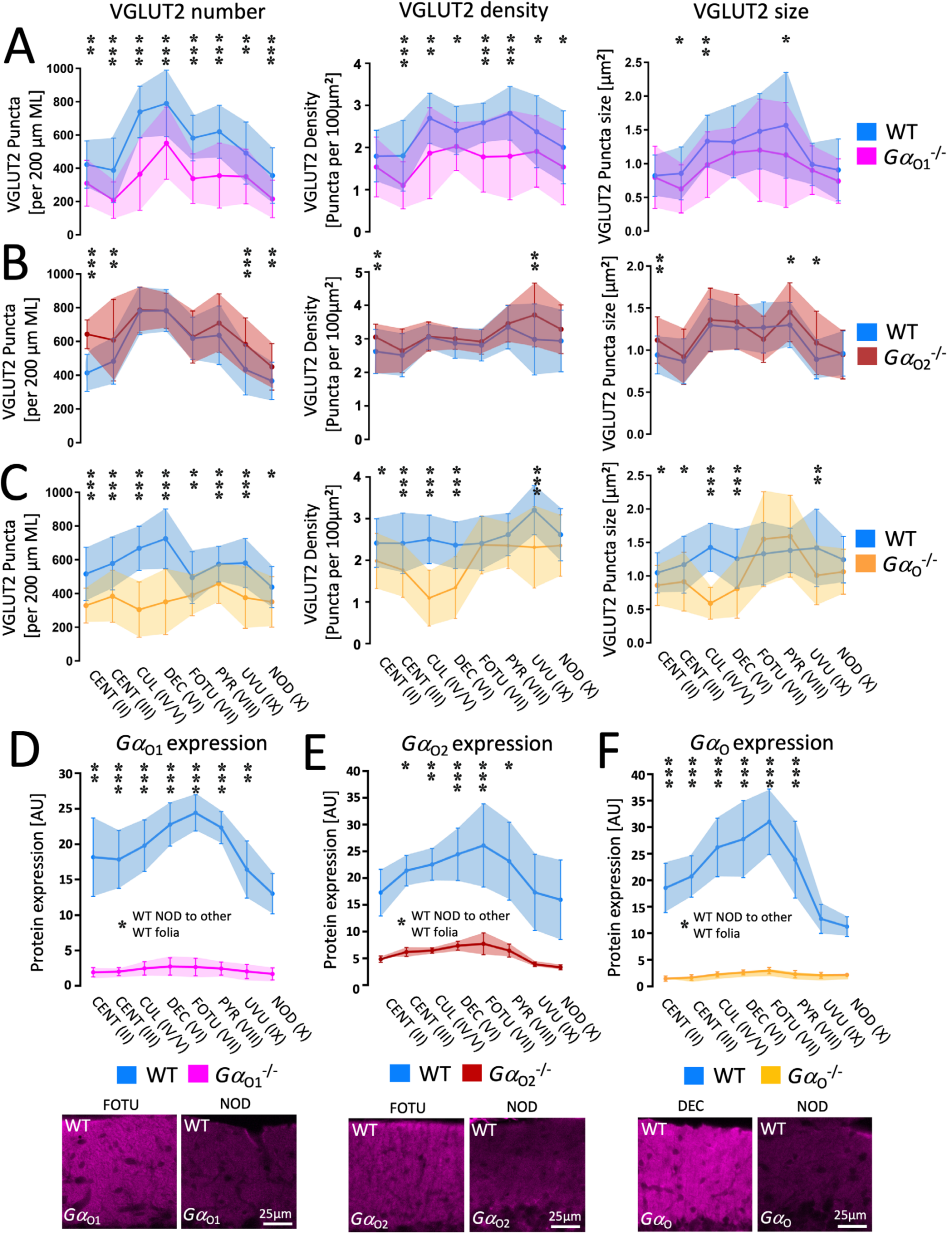
Folia‐specific Gαo subunit expression and determination of Gα_o1_ and Gα_o2_ knockout effects on VGLUT2‐positive synaptic contacts within the cerebellar cortex. Displayed are the effects of Gα_o1_, Gα_o2_, and Gα_o_ knockout on the VGLUT2‐positive synaptic contacts in the molecular layer of the individual folia of the vermis. The same analysis as summarized in Figure 2 for the total molecular layer was conducted for the molecular layer at the level of individual folia. (A) Folia‐specific Gα_o1_ knockout effects were observed for the absolute number (left panel), density (middle panel) and size of VGLUT2 puncta (right panel). (B) Folia‐specific Gα_o2_ knockout effects on the same parameters of VGLUT2 contacts as shown in (A). (C) Folia‐specific Gα_o2_ knockout effects on the same parameters of VGLUT2 contacts as shown in (A). (D) Expression of Gα_o1_ in the individual folia. Cerebellar wild type sections were incubated with the Gα_o1_ antibody and protein expression was quantified for the molecular layer of the individual lobules. The knockout is shown for control. See representative images. (E) The same analysis was performed for the expression of Gα_o2_. See representative images. (F) Analysis of the combined expression of both isoforms by the Gα_o_ antibody confirmed the results observed with the subtype‐specific antibodies. See representative images. Data show means ± SD, *N* = 3–4 animals each WT and knockout, 6–8 sections per animal and genotype. *p ≤ 0.05; **p ≤ 0.01; ***p ≤ 0.001.

We also analyzed these parameters for all knockout strains with respect to the individual folia. The number of synaptic contacts was found to be reduced in all folia of the Gα_o1_ knockout with a maximum effect size of 50% in the culmen (Figure 3A). This was reflected by a reduced density in all folia but the central lobule II (at a maximum of −36.3% in the pyramis, Figure 3A). The size of the VGLUT2 puncta was smaller in 3 folia of the Gα_o1_^−/−^ animals, with the knockout effect being most pronounced at −27.6% also in the pyramis (Figure 3A). With respect to the Gα_o2_ knockout, higher numbers were detected in four folia of the knockout (most pronounced in the central lobule II at +52.6%, Figure 3B) that contributed to the overall effect. The density was only affected in a comparable positive fashion in two folia (central lobule II and uvula, at a maximum of +15.5% in central lobule II, Figure 3B). Similarly, the size was increased in the central lobule II, pyramis, and uvula (to the most at +22% in the central lobule II, Figure 3B).

When Gα_o_ double knockout effects were addressed, significant reductions in the number of puncta per ROI were observed in all folia (at a maximum of −54.4% in the culmen, Figure 3C). In five folia, the VGLUT2 density was lower in the knockout, exhibiting the strongest reduction again in the culmen (−56%, Figure 3C). A similar picture appeared when looking at the VGLUT2 puncta size decreased in five folia with the most prominent effect also in the culmen (−58.5%, Figure 3C). To address the question of whether the differential effects of G protein subunit knockouts on the synaptic makeup visualized by VGLUT2 stainings were also reflected by different expression levels of G*α*_o1_ and G*α*_o2_ in the individual lobules, we performed an expression analysis of the two subunits in the molecular layer by immunofluorescence methods. Indeed, differences in protein expression were detected for G*α*_o1_, peaking in the fotulum‐tuber‐vermis and showing the lowest expression in the nodulus (−48% nodulus vs. fotulum‐tuber‐vermis, Figure 3D). A similar picture emerged for G*α*_o2_, again with the highest expression in the fotulum‐tuber‐vermis and the lowest in the nodulus (−39% nodulus vs. fotulum‐tuber‐vermis, Figure 3E). Highest expression in the fotulum‐tuber‐vermis and lowest in the nodulus was confirmed by the antibody recognizing both isoforms (−63% nodulus vs. fotulum‐tuber‐vermis, Figure 3F).

**FIGURE 4:**
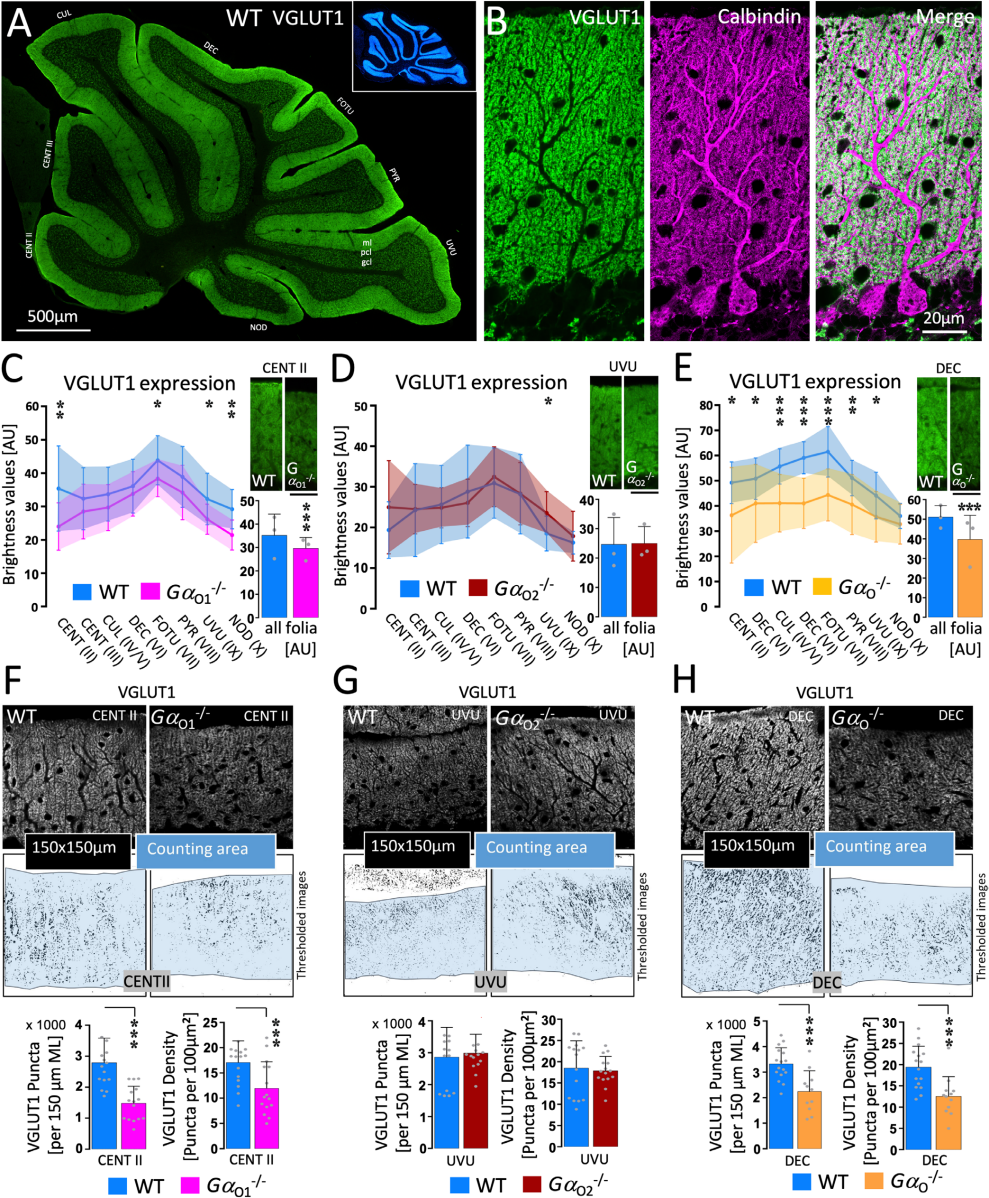
Gα_o1_ and Gα_o2_ differentially affect the number of VGLUT1 contacts within the cerebellar cortex. Immunohistochemical analysis of the vesicular glutamate transporter 1 (VGLUT1) expression in the adult wild type cerebellum as shown in a midsagittal section. A very dense immune signal was detected in the molecular layer. In addition, the cerebellar glomeruli of the granule cell layer (gcl), were marked (see inset for Dapi staining). (B) Double staining for VGLUT1 and Calbindin. In the molecular layer, the VGLUT1 transporter signal forms a dense synaptic carpet sparing the main Purkinje cell dendrite branches. (C) Expression of VGLUT1 in the individual folia. Cerebellar wild type and Gα_o1_^−/−^ sections were incubated with a VGLUT1 antibody and protein expression was quantified for the molecular layer of the individual lobules and expressed as brightness values. See representative images. Scale bar 50 μm. Expression levels of VGLUT1 averaged over all folia are given in the bar chart. (D) The same analysis was performed for wild type and Gα_o2_^−/−^ cerebella. See representative images. Scale bar 50 μm. Expression levels of VGLUT1 averaged over all folia are given in the bar chart. (E) Expression of VGLUT1 in the individual folia of wild type and Gα_o_^−/−^ cerebella. See representative images. Scale bar 50 μm. Expression levels of VGLUT1 averaged over all folia are given in the bar chart. (F) Analysis of the effects of Gα_o1_ knockout on the VGLUT1 staining pattern in the central lobule II of wild type and Gα_o1_^−/−^ mice used to quantify individual VGLUT1 puncta. Exemplary thresholded images are shown used for analysis in the respective regions of interest (counting area, shown in transparent blue). (G) Analysis of the effects of Gα_o2_ knockout on the VGLUT1 staining pattern in the uvula of wild type and Gα_o2_^−/−^ mice as shown in (F). (H) Analysis of the effects of Gα_o_ knockout on the VGLUT1 staining pattern in the declive of wild type and Gα_o_^−/−^ mice as shown in (F) and (G). (B, F–H) Confocal imaging. Data show means ± SD with individual animal means or individual sections depicted in bar charts, *N* = 3 animals each WT and knockout. 5–6 sections per animal and genotype. *p ≤ 0.05, **p ≤ 0.01, ***p ≤ 0.001.

Then, we analyzed alterations caused by G*α*_o_ subunit knockouts on the distribution of another type of excitatory synapses, the VGLUT1‐positive synaptic connections established by the parallel fibers contacting the Purkinje cell dendrites. These synapses represent the majority of synaptic contacts in the cerebellar molecular layer (Napper and Harvey 1988). In addition to staining of the cerebellar glomeruli in the granule cell layer, expression of VGLUT1 presents as a very dense synaptic carpet within the molecular layer sparing the main Purkinje cell dendritic tree (Figure 4A,B). Given the dense distribution of VGLUT1 signals in the molecular layer, we in this case performed a brightness analysis to better compare the expression of this transporter between the individual lobes as primary readout. In the Gα_o1_ wild type, VGLUT1 expression peaked in the fotulum‐tuber‐vermis and was lowest in the nodulus with significant reductions in Gα_o1_^−/−^ cerebella observed in four lobules (on average −17.3%, Figure 4C). Expression of VGLUT1 in the Gα_o2_ wild type again showed the basic principle expression pattern between folia, and knockout of Gα_o2_ resulted in a positive effect in the uvula only (+27.2%, Figure 4D). A comparison between Gα_o_ wild type and knockout cerebella showed significant negative effects elicited by the knockout in all folia but the nodulus (on average −23%, Figure 4E). For selected lobules showing the strongest effects on VGLUT1 expression by the respective knockouts, we again performed quantification of individual VGLUT1‐positive puncta. In the central lobule II, a significant reduction of the total number (−47.1%) and density (−29.4%) of puncta was observed in Gα_o1_^−/−^ cerebella compared to the wild type (Figure 4F). Between the uvulae of wild type and Gα_o2_^−/−^ cerebella brightness differences were detected, the individual analysis, however, was unable to show differences in both number or density of puncta (Figure 4G). For the double knockout, on the other hand, a significant reduction of the total number (−36.6%) and the density (−34.2%) of puncta was confirmed in the declive of Gα_o_^−/−^ cerebella compared to the wild type (Figure 4H).

### 3.3 Effects of Gα_o_ Splice Isoform Knockouts on Inhibitory Synaptic Contacts in the Cerebellar Cortex

**FIGURE 5:**
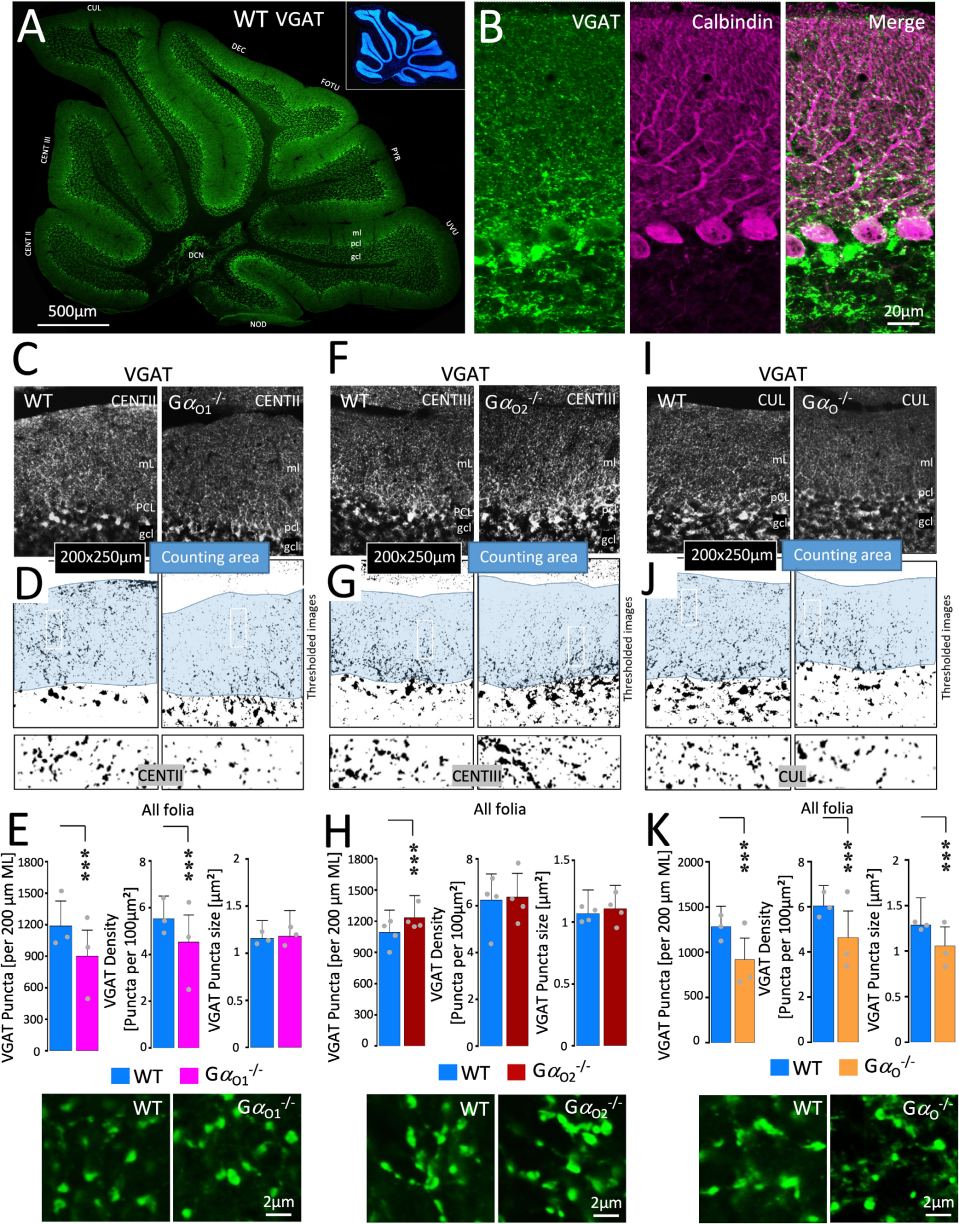
Differential effects of single Gα_o1_, Gα_o2_, and double Gα_o1_/Gα_o2_ knockout on the number and size of VGAT‐positive synaptic contacts in the molecular layer. (A) Immunohistochemical analysis of the vesicular GABA transporter (VGAT) expression in the adult wild type cerebellum as shown in a midsagittal section. A strong immune signal was detected at the Purkinje cell somata, in the cerebellar glomeruli of the granule cell layer (gcl), together with a punctuate pattern in the molecular layer (ml) and the deep cerebellar nuclei (DCN, see inset for Dapi staining). (B) Double staining for Calbindin to visualize Purkinje cell dendrite morphology and VGAT. In the molecular layer, the VGAT signal is detected on all segments of the Purkinje cell dendrites. (C) Analysis of the effects of Gα_o1_ knockout on the VGAT staining pattern in the molecular layer of the cerebellum. Regions of the molecular layer depicting VGAT signals in the central lobule II of wild type and Gα_o1_^−/−^ mice were used to quantify the VGAT puncta. (D) Exemplary thresholded images of the regions are shown used for analysis in the respective regions of interest (counting area, shown in transparent blue, see insets for magnification). (E) Quantification of VGAT puncta in the counting area (left panel), their density (middle panel), and size (right panel). See higher magnification confocal imaging for details of VGAT signals. (F) Analysis of the effects of Gα_o2_ knockout on the VGAT staining pattern in the molecular layer of the cerebellum. Regions of the molecular layer depicting VGAT signals in the central lobule III of wild type and Gα_o2_^−/−^ mice were used to quantify the VGAT puncta. (G) Exemplary thresholded images of the regions are shown used for analysis in the respective regions of interest (counting area, shown in transparent blue, see insets for magnification). (H) Quantification of VGAT puncta in the counting area (left panel), density (middle panel), and size (right panel). See higher magnification confocal imaging for details of VGAT signals. (I) Analysis of the effects of Gα_o1_ and Gα_o2_ double knockout on the VGAT staining pattern in the molecular layer of the cerebellum. Regions of the molecular layer depicting VGAT signals in the culmen of wild type and Gα_o_^−/−^ mice were used to quantify the VGAT puncta. (J) Exemplary thresholded images of the regions are shown used for analysis in the respective regions of interest (counting area, shown in transparent blue, see insets for magnification). (K) Quantification of VGAT puncta in the counting area (left panel), their density (middle panel), and size (right panel). See higher magnification confocal imaging for details of VGAT signals. Bars show means ± SD with individual animal means depicted, *N* = 3 animals each WT/Gα_o1_ KO as well as WT/Gα_o2_ KO, *N* = 4 animals each WT/Gα_o2_ KO; 6–8 sections per animal and genotype. ***p ≤ 0.001.

In addition to the investigation of glutamatergic excitatory contacts, we also addressed the effects of G*α*_o_ splice‐specific knockouts on the formation of inhibitory synapses within the molecular layer by analyzing VGAT (vesicular GABA transporter)‐positive contacts. The distribution of VGAT, indicating the location of inhibitory synapses, typically differs from the VGLUT2 expression pattern in a way that VGAT‐positive synapses are formed at higher numbers within the molecular layer of the cerebellum, spanning from the deepest layer all the way up to the surface area to contact—among other cell types—the distal, finer dendritic branches of the Purkinje cells (Figure 5A,B). Knockout of G*α*_o1_ resulted in an overall reduction of VGAT puncta by 25%, in part due to a reduced density (−18%, Figure 5C–E). The overall size of the VGAT puncta was not affected by the knockout. In the *Gα*_o2_ knockout, overall numbers of VGAT puncta per counting area were moderately enhanced (+13%, Figure 5F–H) while puncta density and size were not altered. In G*α*_o_ mice, all three parameters were downregulated by −31% in counts, −24% in density, and −18% in puncta size (Figure 5I–K).

**FIGURE 6:**
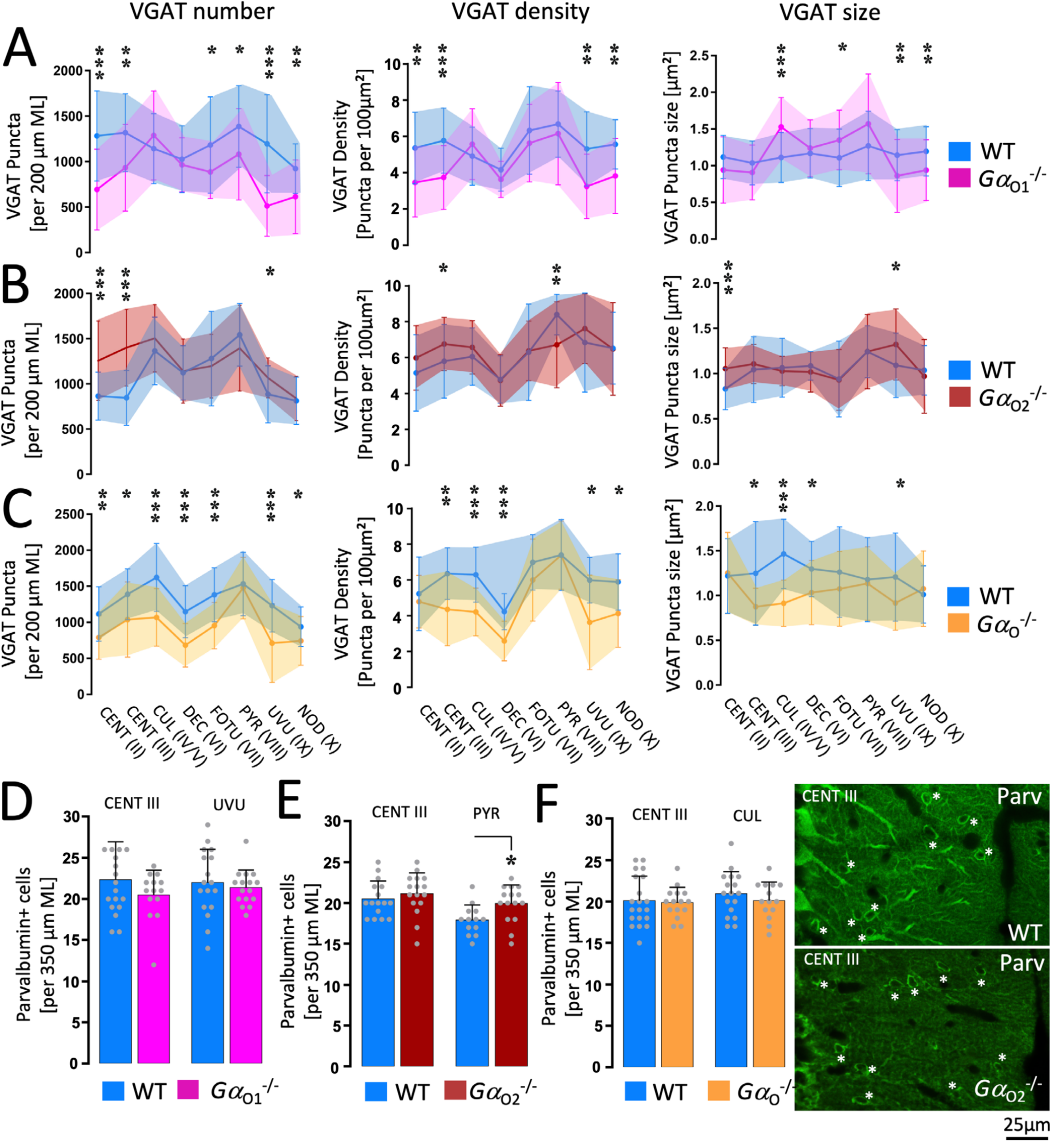
Folia‐specific determination of Gα_o1_ and Gα_o2_ knockout effects on VGAT‐positive synaptic contacts within the cerebellar cortex. Displayed are the effects of Gα_o1_, Gα_o2_, and Gα_o_ knockout on the VGAT‐positive synaptic contacts in the molecular layer of the individual folia of the vermis. (A) Significant reductions appearing in the Gα_o1_ knockout regarding the absolute number (left panel), density (middle panel), and the size of VGAT puncta (right panel) are indicated. (B) The same analysis was performed for the Gα_o2_ knockout. Mainly positive significant effects are indicated. (C) Folia‐specific analysis as in (A) and (B) for the Gα_o_ knockout. Significant reductions are indicated. (D) Number of Parvalbumin‐positive cells within the molecular layer of the lobules CENTIII and uvula in wild type and Gα_o1_ knockout animals. (E) Number of Parvalbumin‐positive cells within the molecular layer of the lobules CENTIII and pyramis in wild type and Gα_o2_ knockout animals. (F) Number of Parvalbumin‐positive cells within the molecular layer of the lobules CENTIII and culmen in wild type and Gα_o_ knockout animals. See representative images for wild type and Gα_o2_^−/−^ cerebella stained with an antibody recognizing Parvalbumin. Asterisks depict cell somata. Data show means ± SD, *N* = 3 animals each WT/Gα_o1_ KO as well as WT/Gα_o2_ KO, *N* = 4 animals each WT/Gα_o2_ KO (VGAT analysis). Parvalbumin counting *N* = 3 animals each condition. *p ≤ 0.05; **p ≤ 0.01; ***p ≤ 0.001.

In detail, the folia‐specific analysis of G*α*_o1_ knockout effects revealed negative effects on the number of inhibitory synapses in six (number per counting area) or four (density) folia. Four individual folia also exhibited positive or negative changes in the puncta size (all Figure 6A). Knockout of G*α*_o2_ resulted in increased counts in three folia (CENTII/III and UVU). Two individual folia also showed mixed effects on density. Puncta size was increased in CENTII and uvula (all Figure 6B). The folia‐specific analysis of the double knockout revealed negative effects in the number of inhibitory synapses in seven, a reduced density in five, and smaller puncta size in four Gα_o_^−/−^ folia contributing to the observed overall reductions (all Figure 6C). To investigate whether changes in the inhibitory synaptic connectivity caused by the respective knockouts resulted from alteration in the number of inhibitory interneurons of the molecular layer, namely basket and stellate cells providing inhibitory input to Purkinje cell dendrites, we addressed this issue. To this end, we quantified the number of individual Parvalbumin‐positive cells in the molecular layer. Parvalbumin is a long established marker of these cell types. We selected two lobules of each knockout strain exhibiting the strongest effects on VGAT synaptic contacts. Counting of Parvalbumin‐positive cells in central lobule III and uvula showed no effects of the G*α*_o1_ knockout (Figure 6D). In the pyramis, but not the central lobule III, knockout of G*α*_o2_ had a slight positive effect (Figure 6E), Neither the central lobule III nor the culmen exhibited effects caused by the knockout of G*α*_o_ (Figure 6F).

**FIGURE 7:**
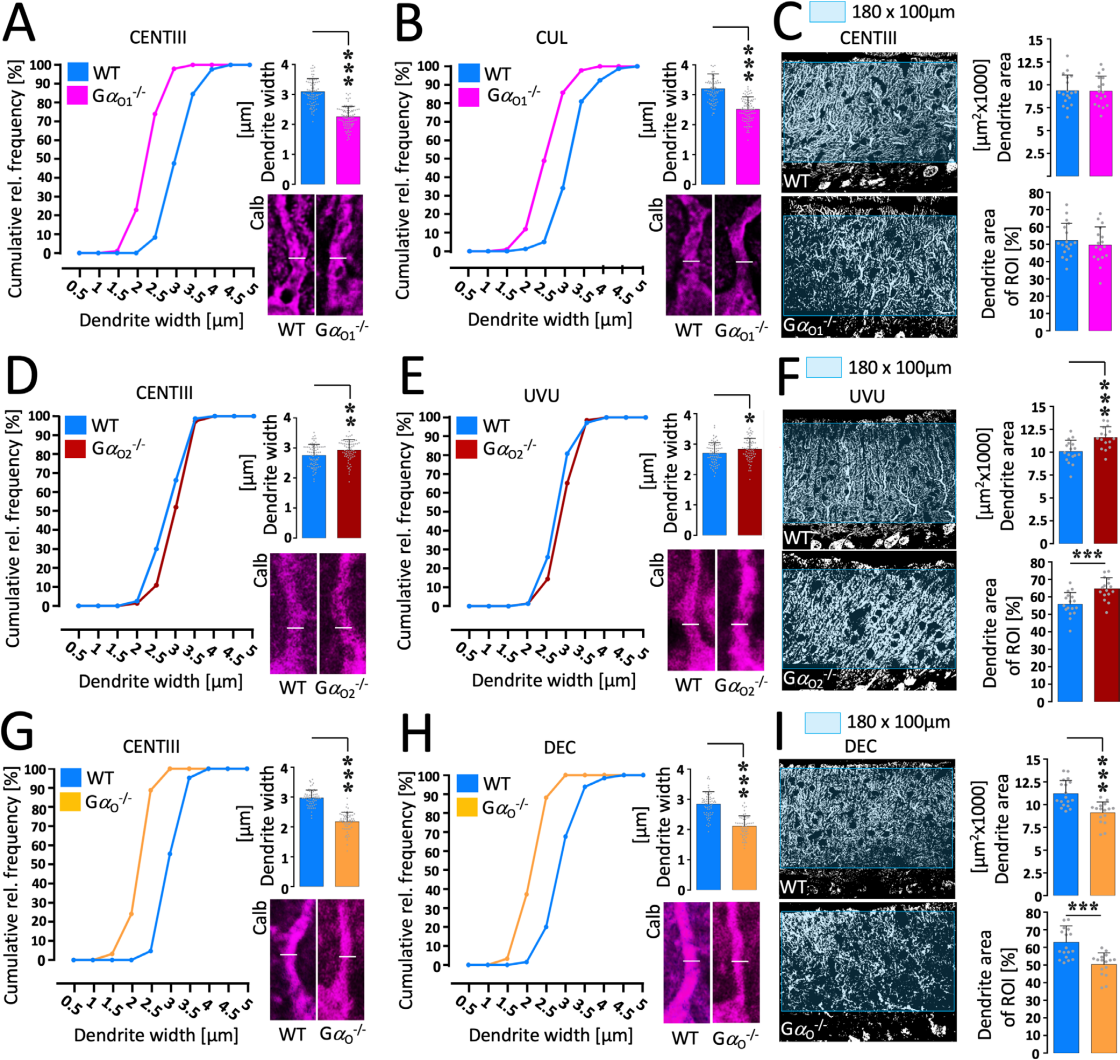
Alterations of Purkinje cell morphology by the knockout of Gα_o1_, Gα_o2_, and the double knockout. (A) Morphometrical analysis of Purkinje cell primary dendrite width in the CENTIII lobule of wild type and Gα_o1_^−/−^ mice. Gα_o1_ knockout effects are displayed as relative cumulative frequency and overall means. White bars in images represent 3 μm. (B) Analysis as shown in (A) performed for primary dendrites in the culmen. Knockout effects are displayed as above. White bars in images represent 3 μm. (C) Analysis of dendrite outgrowth. Regions of interest (ROI) of 180 × 100 μm within the molecular layer of wild type and Gα_o1_^−/−^ cerebella were analyzed and the total Calbindin stained area as well as the proportion of the ROI occupied by the staining were quantified. Thresholded images. (D) Analysis as shown above for the knockout of Gα_o2_ in the CENTIII lobule. White bars in images represent 3 μm. (E) Analysis of effects in the uvula. White bars in images represent 3 μm. (F) Analysis of dendrite outgrowth as performed in (C). Thresholded images. (G) Analysis performed for primary dendrites in the CENTIII lobule of wild type and Gα_o_ knockout mice. (H) Analysis of knockout effects in the declive. (I) Analysis of dendrite outgrowth as performed in (C) and (F). Thresholded images. Data show means ± SD with individual sections depicted in bar charts, *N* = 3 animals each WT and knockout. 5–6 sections per animal and genotype. *p ≤ 0.05, **p ≤ 0.01, ***p ≤ 0.001.

Having observed knockout effects on general cerebellar morphology and synaptic connectivity, we further aimed to decipher whether Purkinje cell morphology is altered by the depletion of G*α*_o1_, G*α*_o2_, or both isoforms. Therefore, we first measured the thickness of the primary dendrite rising from the cell soma into the molecular layer to form its extensive branching. Exemplarily, folia exhibiting the most pronounced effects in the former analyses were chosen. Width measurements of the proximal dendrite stem in the centrale lobule III and culmen showed a reduction in thickness by 24.8% and 21.9%, respectively, in the G*α*_o1_ knockout compared to the wild type (Figure 7A,B). Additionally, the area of the whole dendritic tree within defined regions of 180 μm width and 100 μm height of the molecular layer were measured for the central lobule III. Despite the thinner proximal main dendrites, no significant changes were observed in the knockout (Figure 7C). The same analysis was performed for the G*α*_o2_ knockout and revealed slightly thicker primary dendrites in the centrale lobule III (+6.2%) and the uvula (+5.2%) in the knockout (Figure 7D,E). In line with this, overall dendritic branching was moderately enhanced by the G*α*_o2_ knockout in the uvula looked at exemplarily (+15.1% dendritic area, Figure 7F). Knockout of G*α*_o_ resulted in thinner primary dendrites both in the central lobule III (−26.9%) and the declive (−25.7%) as revealed by the analysis (Figure 7G,H). Again, the overall branching was also reduced in the declive of Gα_o_^−/−^ cerebella (−18.7% dendritic area, Figure 7I).

To sum up, knockout of G*α*_o1_ resulted in a lower number of VGLUT2 and VGLUT1 synaptic contacts to the Purkinje cell dendrites that were also reduced in size compared to the wild type as investigated for VGLUT2. This holds also true for VGAT contacts mainly in terms of their number and density. Depletion of G*α*_o2_ resulted in slightly less pronounced reverse, positive effects on many of the parameters looked at for both excitatory and inhibitory synapses. When both splice isoforms were knocked out, robust negative effects were again observed for VGLUT2, VGLUT1, and VGAT.

Additionally, effects on Purkinje cell morphology were detected that pointed in the same directions.

## 4 Discussion

### 4.1 Knockout Effects on the Cerebellar Anatomy

In the current study we were able to show that knockout of either G*α*_o1_ alone or the double knockout of G*α*_o1_ and G*α*_o2_ (G*α*_o_) resulted in moderate, yet significant, negative effects on the overall cerebellar size. A more in‐depth analysis of the effects on the cortical layers of the vermis region demonstrated that the overall effects observed represented a net outcome of a varying effect strength on the individual lobules, or folia, of the cerebellum. In terms of molecular layer thickness, the anteriorly oriented central lobules II and III exhibited the strongest reductions. Generally, the granule cell layer was less affected by the knockout of G*α*_o1_ or complete G*α*_o_. Single G*α*_o2_ knockout, on the other hand, resulted in moderate positive effects on the overall cerebellar size and individual thickness or area parameters of many of the folia. Morphological analysis of Purkinje cell dendritic growth detected negative effects caused by the absence of G*α*_o1_ while knockout of G*α*_o2_ rather had an opposite outcome. These effects might very well cause or at least contribute to thickness alteration of the molecular layer. Our findings correlate with earlier results demonstrating cerebellar hypoplasia in complete G*α*_o_ knockout animals, caused by a reduced Purkinje cell arborization in the molecular layer (Cha et al. 2019). The current study extends and further deciphers these data in identifying G*α*_o1_ as the responsible subunit for the observed cerebellar hypoplasia. Knockout of G*α*_o2_, on the other hand, resulted in moderate, opposed, positive effects on cerebellar growth. Given that the double knockout also displayed negative growth effects, it appears that G*α*_o1_ function is dominant over G*α*_o2_, and loss of G*α*_o1_ signaling cannot be compensated for by the depletion of G*α*_o2_ function at the same time. Opposing growth‐promoting (knockout of G*α*_o2_) or ‐reducing effects (knockout of G*α*_o1_) were also shown by our group for cultured hippocampal neurons, together with negative effects of G*α*_o1_ depletion on the outgrowth and synaptic connectivity of the hippocampal mossy fiber tract (Baron et al. 2013; Höltje et al. 2025). On the functional level, Gα_o1_ knockout mice differ from Gα_o2_ knockout mice in the striatal dopaminergic system and exhibit motor deficits compared to their wild type littermates as shown by rotarod behavioral experiments (Baron et al. 2018). The overriding importance of G*α*_o1_ over G*α*_o2_ seems to be even more pronounced in the cerebellum than in the hippocampal mossy fiber tract formation, since the double knockout was able to rescue the negative single G*α*_o1_ knockout effects on mossy fiber tract formation, but not the cerebellar hypoplasia, as shown in the current study. Generally, both Gα_o_ subunits are expressed at neuronal growth cones and involve a multitude of effectors to regulate axon outgrowth (Bromberg et al. 2008; Sharma et al. 2015). More specifically, expression of G*α*_o_ subunits is highly abundant postsynaptically at Purkinje cell dendrites and is proposed to represent a major signal transducer in this, but also other neuronal cell populations of the cerebellum (Roldán‐Sastre et al. 2021). Interaction of Gαo proteins with Purkinje cell protein‐2 (Pcp‐2) acting as nucleotide exchange factor for Gαo has been demonstrated some time ago and has been shown to be involved in stimulating neuronal process outgrowth and differentiation and can also be used as a Purkinje cell marker in recently developed human cerebellar organoids (Luo and Denker 1999; Redd et al. 2002; Atamian et al. 2024) and nonfunctional Pcp‐2 mutant mice also display moderate cerebellar hypoplasia (Iscru et al. 2009). In this context, lack of G*α*_o1_ or G*α*_o2_ might differentially interfere with Pcp‐2 dependent Purkinje cell dendrite formation, although its precise role in the morphogenesis of this cell type is largely unknown.

### 4.2 Knockout Effects on Excitatory and Inhibitory Synaptic Contacts

In light of the increasing evidence that the individual folia separated by the cerebellar fissures serve as platforms for organizing intracerebellar circuits, and mapping of circuits and physiological investigations have attributed particular sensory‐motor tasks to certain folia (Sotelo and Chédotal 2005; Sillitoe and Joyner 2007) synaptic analyses were also performed in a folia‐specific analysis.

In the majority of the folia, knockout of either *Gα*_o1_ alone or both subunits resulted in significant reductions in the number and in some cases the size of VGLUT2‐positive contacts in the molecular layer. Likewise, expression of VGLUT1 was negatively affected in both knockout strains. Somewhat less pronounced, similar effects on VGAT could be observed. Again, knockout of *Gα*_o2_ moderately stimulated mainly VGLUT2 and VGAT expression, while VGLUT1 was only affected to a minor extent. Reduced or increased numbers mostly resulted from a combination of effects on synaptic density and alterations in the molecular layer thickness. We refrained from analyzing effects in the granule cell layer, since the anatomical complexity of the cerebellar glomeruli makes a quantitative analysis much more challenging and would not include contacts to Purkinje cell dendrites. The current study shows that also in the process of establishing both climbing and parallel fiber contacts, as well as inhibitory synapses within the molecular layer, *Gα*_o1_ functions seem to oppose *Gα*_o2_‐mediated signaling and to dominate over *Gα*_o2_, since the double knockout was unable to restore the wild type values. As already stated for the observed cerebellar hypoplasia, reductions in the number and size of VGLUT2‐positive climbing fiber contacts had been demonstrated in double knockout animals (Cha et al. 2019). In this context, the authors showed a reduced Purkinje cell dendritic arborization and atrophied spines as the underlying mechanisms. Changes in *Gα*_o_ knockout spine morphology go along with the observation that *Gα*_o_ is highly expressed postsynaptically at excitatory synapses (Roldán‐Sastre et al. 2021). Our morphological data propose the same alterations in Purkinje cell dendrite morphology for the observed *Gα*_o1_ knockout effects and an increased dendritic growth in *Gα*_o2_ knockout animals as already shown for cultured neurons (Baron et al. 2013; Höltje et al. 2025). Interestingly, a monoallelic GNAO1 (the gene that encodes for both splice isoforms) loss of function mutation was described in mice with movement disorder that exhibit reduced inhibitory synaptic input to Purkinje cells in expressing significantly lower frequencies of spontaneous and miniature inhibitory postsynaptic currents (Feng et al. 2022). Accompanied with these electrophysiological findings was a moderately reduced number in molecular layer GABAergic interneurons. Our data do not support a reduced number of inhibitory interneurons caused by the depletion of *Gα*_o1_, but rather point to a reduced Purkinje cell dendritic complexity providing fewer postsynaptic contact sites as a possible mechanism for decreased synaptic contacts in general and specifically in the case of the observed reduced VGAT signals in *Gα*_o1_ and *Gα*_o_ knockout mice. One aspect, however, we cannot fully rule out to contribute to an altered number of VGAT‐positive contacts are changes in interneuron morphology and synaptic connections between individual stellate cells or stellate and basket cells. This is an issue very hard to address given that there is no specific marker for these cell types leaving Purkinje cell dendrites unstained. The effects on VGLUT1/2‐positive glutamatergic parallel and climbing fiber synapses and GABAergic inputs were not evenly distributed between the individual folia. In line with this, expression levels of *Gα*_o1_ and *Gα*_o2_ varied between individual folia. Generally, expression of both subunits was rather low in the posteriorly oriented folia such as the uvula and the nodulus. For some parameters like VGLUT2 and VGAT number, density, and size, as well as VGLUT1 expression levels knockout effects were the lowest in this lobule, especially in the doble knockout. In humans, a growing body of evidence has emerged that besides its relatively uniform anatomy, a strong functional parcellation of the cerebellum exists that supports the idea that the same underlying circuit implements functionally distinct computations in motor and even cognitive tasks (Diedrichsen et al. 2019; King et al. 2019). In this context, all spinocerebellar mossy fiber afferents project specifically to lobules I‐V (CENTII/III and culmen) and VIII/anterior IX (pyramis and uvula) of the vermis (Sotelo and Chédotal 2005; Sillitoe and Joyner 2007). In terms of various neurodegeneration models, folia X (nodulus) has been shown to be more resistant to Purkinje cell degeneration than all other folia, further exemplifying heterogenity of the cerebellar folia also in pathophysiology (Hernández‐Pérez et al. 2025). Therefore, depletion of the respective *Gα*_o_ subunits might include folia‐specific effects that differentially contribute to the observed phenotype, for example, motor deficits that especially *Gα*_o1_ knockout mice exhibit.

Taken together, our study demonstrates that Gα_o1_ stimulates development of the cerebellum, most likely by fostering Purkinje cell growth and formation of both excitatory and inhibitory synaptic connections. Its knockout results in cerebellar hypoplasia and diminished establishment of VGLUT1/2 and VGAT‐positive synaptic connections in the molecular layer as demonstrated in a folia‐specific analysis. Opposing effects on dendritic outgrowth and establishment of synapse formation emerged by depletion of Gα_o2_, thereby emphasizing the antagonistic function of these two widely expressed Gα_o_ subunits for cerebellar development. When both splice variants are deleted, the Gα_o1_ effects dominate over Gα_o2_ effects. Future studies will have to address the exact signaling pathways differentially stimulated by either subtype.

## Author Contributions

**Anton Wolkowicz:** investigation, writing – review and editing, data curation, methodology, visualization. **Markus Höltje:** conceptualization, investigation, writing – original draft, validation, writing – review and editing, supervision, visualization. **Gudrun Ahnert‐Hilger:** conceptualization, validation, writing – review and editing, funding acquisition.

## Funding

This work was supported by grants of the Deutsche Forschungsgemeinschaft (DFG) to G.A.‐H. (GAH 67/10–1).

## Conflicts of Interest

The authors declare no conflicts of interest.

## Supporting information


**Figure S1:** Verification of Gαo subunit‐specific knockouts by genotyping and Western blotting (A) Exemplary genotyping of wild type and single Gα_o1_, Gα_o2_, and double Gα_o1/2_ (Gα_o_) homozygous knockout animals. Genomic template DNA was isolated from ear punches and amplified by PCR. In the wild type, DNA of all three subtypes was clearly detectable but absent in homozygous mice. Neomycin (Neo) cassette DNA confirmed knockout. (B) Western blot analysis of Gα_o_ subunit protein expression. Brain homogenates of wild type and Gα_o1_^−/−^, Gα_o2_^−/−^, and Gα_o_^−/−^ mice were stained by antibodies preferentially recognizing Gα_o1_ (upper panel), Gα_o2_ (middle panel), or both Gα_o1_ and Gα_o2_ (lower panel). Vesicular Synaptobrevin (Syb) was used as control protein. Major bands around 40 kDa corresponding to the expected molecular weight were detected in the wild type that were absent in the knockout.
**Figure S2:** Differential effects of single Gαo1, Gαo2, and double Gαo1/Gαo2 knockout on the cortex thickness and area of individual cerebellar folia. (A) Determination of the thickness of the molecular layer in individual cerebellar lobules by mid‐sagittal sections of the vermal region. Knockout of Gαo1 resulted in the reduction of molecular layer thickness in 5 out of 8 investigated lobules, most prominently in the cranial lobules CENTII, CENTIII and CUL. (B) Area size quantification of the molecular layer in individual cerebellar lobules. Knockout of Gαo1 resulted in the size reduction of molecular layer in 6 out of 8 investigated lobules. (C) Determination of the thickness of the granule cell layer in individual cerebellar lobules. Knockout of Gαo1 resulted in the reduction of thickness in the declive only. (D) Knockout of Gαo1 resulted in the size reduction of three folia. (E) Determination of the thickness of the molecular layer in individual cerebellar lobules. Knockout of Gαo2 resulted in increased molecular layer thickness in 7 out of 8 investigated lobules. (F) Area size quantification of the molecular layer in individual cerebellar lobules. Knockout of Gαo2 resulted in thickening of the molecular layer in 5 out of 8 investigated lobules. (G) Granule cell layer thickness was increased in 5 folia by the Gαo2 knockout. (H) Knockout of Gαo2 resulted in the enlargement of four folia areas. (I) Determination of the thickness of the molecular layer in individual cerebellar lobules following knockout of Gαo. Knockout resulted in the reduction of molecular layer thickness in 7 out of 8 investigated lobules, most prominently in the cranial lobules CENTII, CENTIII and DEC. (J) Area size quantification of the molecular layer in individual cerebellar lobules. Knockout of Gαo resulted in the size reduction of molecular layer in 6 out of 8 investigated lobules. (K) Determination of the thickness of the granule cell layer. Knockout of Gαo resulted in the reduction of thickness in four folia. (L) Knockout of Gαo resulted in a reduced folia size of three folia. CENT, central lobule; CUL, culmen; DEC, declive; FOTU, fotulus/nodulus; NOD, nodulus; PYR, pyramis; UVU, uvula.
**Data show:** means ± SD, *N* = 4 animals each WT and knockout, except for 3 animals in Gαo2 WT, 6–8 sections per animal and genotype. *p ≤ 0.05; **p ≤ 0.01; ***p ≤ 0.001.

## Data Availability

The data that support the findings of this study are available from the corresponding author upon reasonable request.
