# Supplementary material for: Splice Type‐Specific Effects of Gαo Subunits on Cerebellar Anatomy and Synapse Formation
Source: J Neurochem. 2026 Jul 2;170(7):e70512. doi: 10.1111/jnc.70512 (PMC13324400; doi:10.1111/jnc.70512)
Supplement: Supplementary file 1 — Figure S1: Verification of Gαo subunit‐specific knockouts by genotyping and Western blotting (A) Exemplary genotyping of wild type and single Gαo1, Gαo2, and double Gαo1/2 (Gαo) homozygous knockout animals. Genomic template DNA was isolated from ear punches and amplified by PCR. In the wild type, DNA of all three subtypes was clearly detectable but absent in homozygous mice. Neomycin (Neo) cassette DNA confirmed knockout. (B) Western blot analysis of Gαo subunit protein expression. Brain homogenates of wild type and Gαo1 −/−, Gαo2 −/−, and Gαo −/− mice were stained by antibodies preferentially recognizing Gαo1 (upper panel), Gαo2 (middle panel), or both Gαo1 and Gαo2 (lower panel). Vesicular Synaptobrevin (Syb) was used as control protein. Major bands around 40 kDa corresponding to the expected molecular weight were detected in the wild type that were absent in the knockout. Figure S2: Differential effects of single Gαo1, Gαo2, and double Gαo1/Gαo2 knockout on the cortex thickness and area of individual cerebellar folia. (A) Determination of the thickness of the molecular layer in individual cerebellar lobules by mid‐sagittal sections of the vermal region. Knockout of Gαo1 resulted in the reduction of molecular layer thickness in 5 out of 8 investigated lobules, most prominently in the cranial lobules CENTII, CENTIII and CUL. (B) Area size quantification of the molecular layer in individual cerebellar lobules. Knockout of Gαo1 resulted in the size reduction of molecular layer in 6 out of 8 investigated lobules. (C) Determination of the thickness of the granule cell layer in individual cerebellar lobules. Knockout of Gαo1 resulted in the reduction of thickness in the declive only. (D) Knockout of Gαo1 resulted in the size reduction of three folia. (E) Determination of the thickness of the molecular layer in individual cerebellar lobules. Knockout of Gαo2 resulted in increased molecular layer thickness in 7 out of 8 investigated lobules. (F) Area size quantification o [file JNC-170-e70512-s001.pdf]

Supplementary Material

Splice type-specific effects of Gαo subunits on cerebellar anatomy and synapse formation

Markus Höltje, Anton Wolkowicz, and Gudrun Ahnert-Hilger

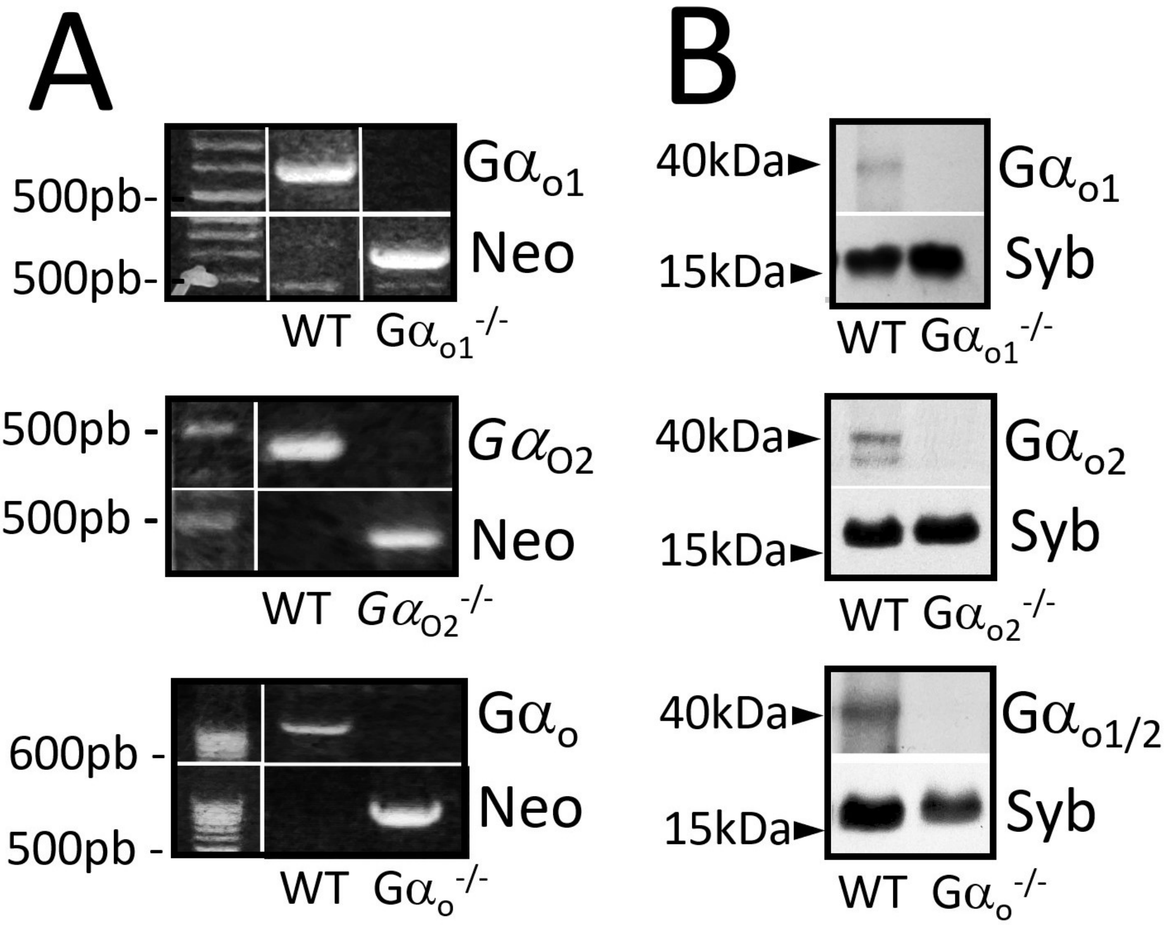

Supplemental Figure S1

Verification of Gαo subunit-specific knockouts by genotyping and Western blotting

A) Exemplary genotyping of wild type and single $G\alpha_{o1}$, $G\alpha_{o2,}$ and double $G\alpha_{o1/2}$ ($G\alpha_{o}$) homozygous knockout animals. Genomic template DNA was isolated from ear punches and amplified by PCR. In the wild type, DNA of all three subtypes was clearly detectable but absent in homozygous mice. Neomycin (Neo) cassette DNA confirmed knockout. B) Western blot analysis of $G\alpha_{o}$ subunit protein expression. Brain homogenates of wild type and $G\alpha_{o1}^{-/-}$, $G\alpha_{o2}^{-/-}$, and $G\alpha_{o}^{-/-}$ mice were stained by antibodies preferentially recognizing $G\alpha_{o1}$ (upper panel), $G\alpha_{o2}$ (middle panel), or both $G\alpha_{o1}$ and $G\alpha_{o2}$ (lower panel). Vesicular Synaptobrevin (Syb) was

used as control protein. Major bands around 40kDa corresponding to the expected molecular weight were detected in the wild type that were absent in the knockout.

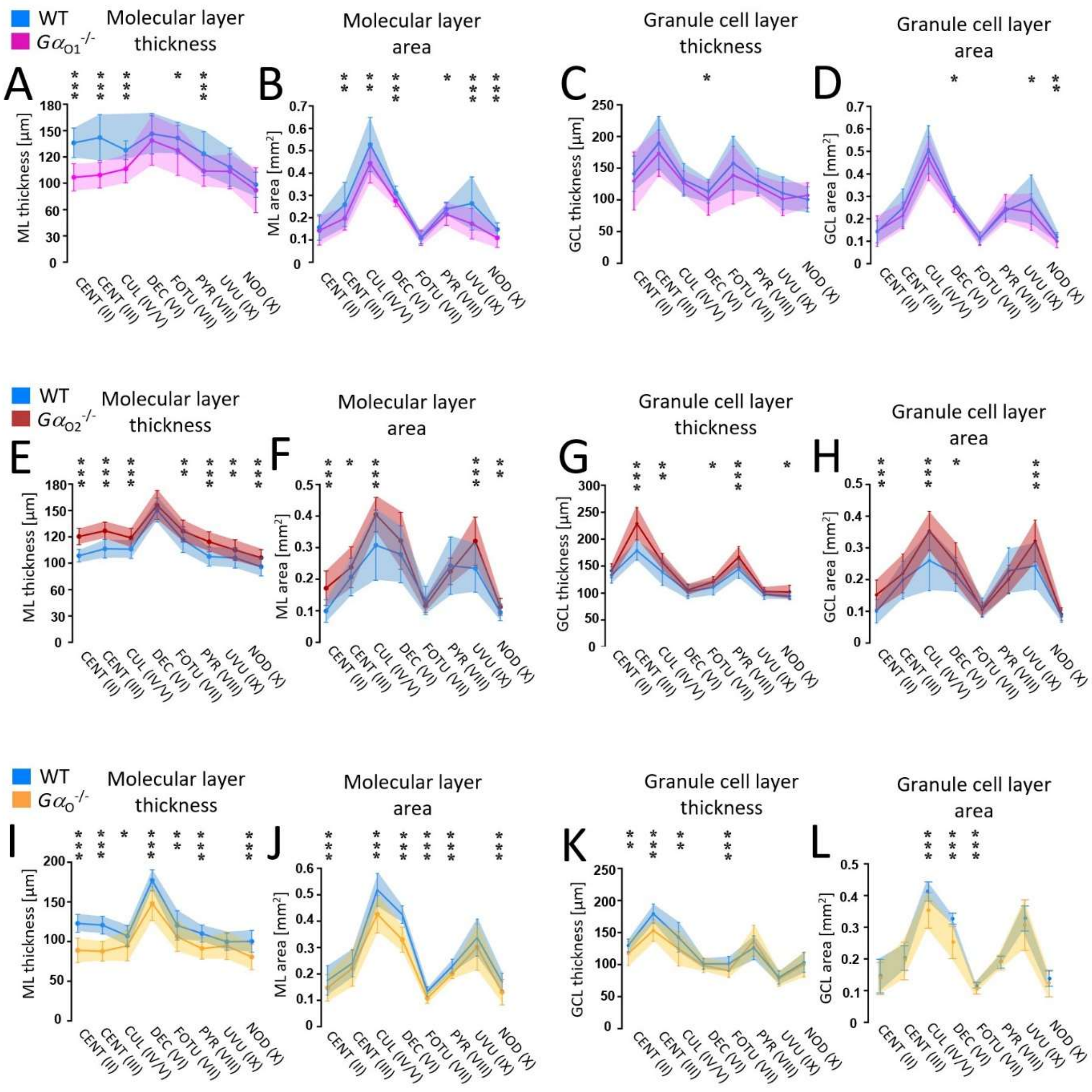

Supplemental Figure S2

Differential effects of single $G\alpha_{o1}$, $G\alpha_{o2}$, and double $G\alpha_{o1/}$ $G\alpha_{o2}$ knockout on the cortex thickness and area of individual cerebellar folia

A) Determination of the thickness of the molecular layer in individual cerebellar lobules by mid-sagittal sections of the vermal region. Knockout of $G\alpha_{o1}$ resulted in the reduction of molecular layer thickness in 5 out of 8 investigated lobules, most prominently in the cranial lobules CENTII, CENTIII and CUL. B) Area size quantification of the molecular layer in individual cerebellar lobules. Knockout of $G\alpha_{o1}$ resulted in the size reduction of molecular layer in 6 out of 8 investigated lobules. C) Determination of the thickness of the granule cell layer in individual cerebellar lobules. Knockout of $G\alpha_{o1}$ resulted in the reduction of thickness in the declive only. D) Knockout of $G\alpha_{o1}$ resulted in the size reduction of three folia. E) Determination of the thickness of the molecular layer in individual cerebellar lobules. Knockout of $G\alpha_{o2}$ resulted in increased molecular layer thickness in 7 out of 8 investigated lobules. F) Area size quantification of the molecular layer in individual cerebellar lobules. Knockout of $G\alpha_{o2}$ resulted in thickening of the molecular layer in 5 out of 8 investigated lobules. G) Granule cell layer thickness was increased in 5 folia by the $G\alpha_{o2}$ knockout. H) Knockout of $G\alpha_{o2}$ resulted in the enlargement of four folia areas. I) Determination of the thickness of the molecular layer in individual cerebellar lobules following knockout of $G\alpha_{o.}$ Knockout resulted in the reduction of molecular layer thickness in 7 out of 8 investigated lobules, most prominently in the cranial lobules CENTII, CENTIII and DEC. J) Area size quantification of the molecular layer in individual cerebellar lobules. Knockout of $G\alpha_o$ resulted in the size reduction of molecular layer in 6 out of 8 investigated lobules. K) Determination of the thickness of the granule cell layer. Knockout of $G\alpha_o$ resulted in the reduction of thickness in four folia. L) Knockout of $G\alpha_o$ resulted in a reduced folia size of three folia. CENT central lobule; CUL culmen: DEC declive; FOTU fotulus/nodulus; PYR pyramis; UVU uvula; NOD nodulus

Data show means ± SD, N = 4 animals each WT and knockout, except for 3 animals in $G\alpha_{o2}$ WT), 6-8 sections per animal and genotype. *P≤0,05; **P≤0,01; ***P≤0,001

Supplemental Figure 1B
upper panel

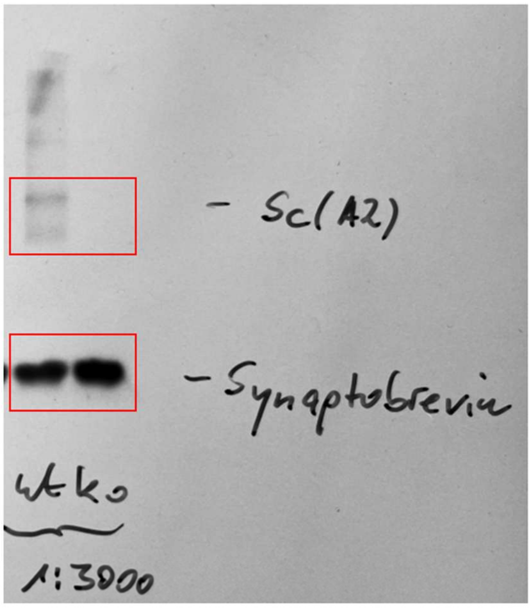

Supplemental Figure 1B
middle panel

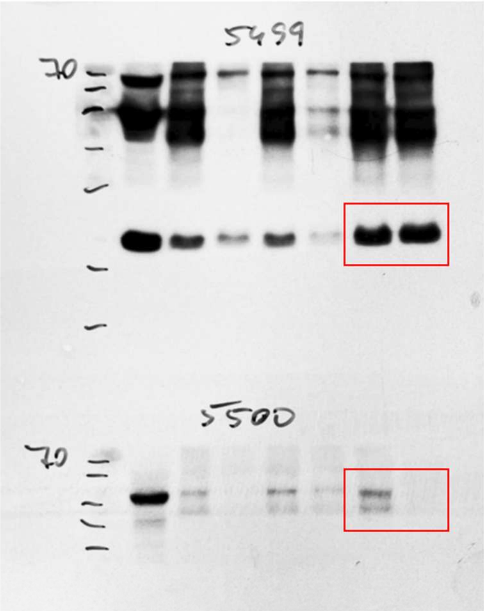

Syb Ab

101.2 Ab

WT Gαo KO WT Gαo1 KO WT Gαo2 KO

Supplemental Figure 1B
lower panel

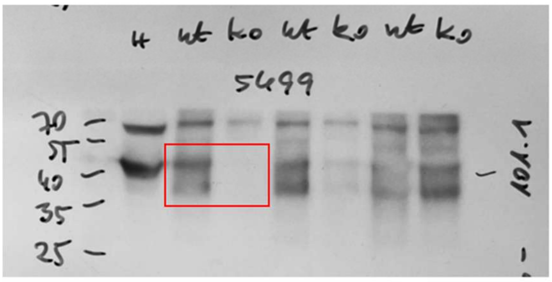

101.1 Ab

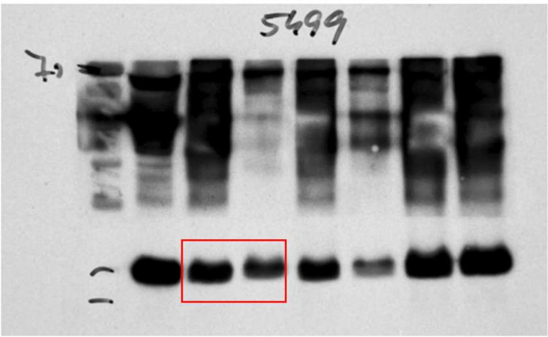

Syb Ab

WT Gαo KO WT Gαo1 KO WT Gαo2 KO

WT vs. Gαo1 KO

unpaired							
two tailed t-test	**Cerebellum area**		**Length**	**Height**		**ML Area**	**GCL Area**
p-value	0,0006		2,76E-06	0,00011		0,1365	0,6083
Degree of Freedom	df 58		df 62	df 51		df 62	df 62
T-Value	2,0017175		1,9989715	2,0095752		1,9989715	1,9989715

unpaired	**Molecular layer thickness**							
two tailed t-test	CENTII	CENT III	CUL	DEC	FOTU	PYR	UVU	NOD
p-value	1,36E-13	1,83E-10	3,89E-08	0,269	0,01658	0,00063	0,36194	0,2348
Degree of Freedom	df 58	df 58	df 58	df 58	df 58	df 58	df 58	df 58
T-Value	2,0017175	2,0017175	2,0017175	2,0017175	2,0017175	2,0017175	2,0017175	2,0017175

unpaired	**Molecular layer Area**							
two tailed t-test	CENTII	CENT III	CUL	DEC	FOTU	PYR	UVU	NOD
p-value	0,3588	0,0046	0,007	3,50E-05	0,9535	0,0299	0,0005	0,0002
Degree of Freedom	df 58	df 58	df 58	df 58	df 58	df 58	df 58	df 58
T-Value	2,0017175	2,0017175	2,0017175	2,0017175	2,0017175	2,0017175	2,0017175	2,0017175

unpaired	**Granule Cell layer thickness**							
two tailed t-test	CENTII	CENT III	CUL	DEC	FOTU	PYR	UVU	NOD
p-value	0,2428	0,1014	0,412	0,0483	0,09	0,0809	0,1645	0,2055
Degree of Freedom	df 58	df 58	df 58	df 58	df 58	df 58	df 58	df 58
T-Value	2,0017175	2,0017175	2,0017175	2,0017175	2,0017175	2,0017175	2,0017175	2,0017175

unpaired	**Granule Cell layer Area**							
two tailed t-test	CENTII	CENT III	CUL	DEC	FOTU	PYR	UVU	NOD
p-value	0,7932	0,0973	0,2076	0,0258	0,4619	0,3307	0,0387	0,0076
Degree of Freedom	df 58	df 58	df 58	df 58	df 58	df 58	df 58	df 58
T-Value	2,0017175	2,0017175	2,0017175	2,0017175	2,0017175	2,0017175	2,0017175	2,0017175

		**Gao1 Expression**						
WT NOD against	unpaired							
other WT folia	two tailed t-t	CENTII	CENT III	CUL	DEC	FOTU	PYR	UVU
	p-value	0,0012186	0,0001837	2,457E-07	4,315E-12	2,009E-14	7,041E-10	0,0049849
	Degree of Fre	df 36	df 36	df 36	df 36	df 36	df 36	df 36
	T-Value	2,028094	2,028094	2,028094	2,028094	2,028094	2,028094	2,028094

WT vs. Gαo1 KO

unpaired						
two tailed t-test	**VGLUT2 Count total**		**VGLUT2 Count Density**		**VGLUT2 Count Size**	
p-value	6,9257E-23		4,7049E-14		2,66965E-05	
Degree of Freedom	df 58		df 58		df 58	
T-Value	2,00171748		2,00171748		2,001717484	

unpaired	**VGLUT2 counts**							
two tailed t-test	CENTII	CENT III	CUL	DEC	FOTU	PYR	UVU	NOD
p-value	0,00426522	0,00014821	8,5364E-09	5,14239E-05	1,82543E-07	1,59954E-06	0,0033313	0,00052727
Degree of Freedom	df 58	df 58	df 58	df 58	df 58	df 58	df 58	df 58
T-Value	2,00171748	2,00171748	2,00171748	2,001717484	2,001717484	2,001717484	2,0017175	2,001717484

unpaired	**VGLUT2 density**							
two tailed t-test	CENTII	CENT III	CUL	DEC	FOTU	PYR	UVU	NOD
p-value	0,15636216	0,00043474	0,00101387	0,014623047	9,61564E-05	0,000104327	0,0428566	0,048614296
Degree of Freedom	df 58	df 58	df 58	df 58	df 58	df 58	df 58	df 58
T-Value	2,00171748	2,00171748	2,00171748	2,001717484	2,001717484	2,001717484	2,0017175	2,001717484

unpaired	**VGLUT2 size**							
two tailed t-test	CENTII	CENT III	CUL	DEC	FOTU	PYR	UVU	NOD
p-value	0,80785697	0,02507052	0,00393678	0,191274327	0,13191986	0,045295228	0,3487286	0,117902818
Degree of Freedom	df 58	df 58	df 58	df 58	df 58	df 58	df 58	df 58
T-Value	2,00171748	2,00171748	2,00171748	2,001717484	2,001717484	2,001717484	2,0017175	2,001717484

unpaired						
two tailed t-test	**VGAT Count total**		**VGAT Count Density**		**VGAT Count Size**	
p-value	6,3005E-08		1,4786E-05		0,594514124	
Degree of Freedom	df 46		df 46		df 46	
T-Value	2,0128956		2,0128956		2,012895599	

unpaired	**VGAT counts**							
two tailed t-test	CENTII	CENT III	CUL	DEC	FOTU	PYR	UVU	NOD
p-value	0,00031297	0,00756608	0,3874003	0,494622501	0,02412906	0,017559903	0,000113	0,001039314
Degree of Freedom	df 46	df 46	df 46	df 46	df 46	df 46	df 46	df 46
T-Value	2,0128956	2,0128956	2,0128956	2,012895599	2,012895599	2,012895599	2,0128956	2,012895599

unpaired	**VGAT density**							
two tailed t-test	CENTII	CENT III	CUL	DEC	FOTU	PYR	UVU	NOD
p-value	0,00256498	0,00045924	0,21162027	0,107782066	0,314263222	0,472478711	0,0022283	0,00221695
Degree of Freedom	df 46	df 46	df 46	df 46	df 46	df 46	df 46	df 46
T-Value	2,0128956	2,0128956	2,0128956	2,012895599	2,012895599	2,012895599	2,0128956	2,012895599

unpaired	**VGAT size**							
two tailed t-test	CENTII	CENT III	CUL	DEC	FOTU	PYR	UVU	NOD
p-value	0,16803886	0,2160139	0,0006304	0,377668568	0,046777211	0,11553397	0,0072759	0,007062105
Degree of Freedom	df 46	df 46	df 46	df 46	df 46	df 46	df 46	df 46
T-Value	2,0128956	2,0128956	2,0128956	2,012895599	2,012895599	2,012895599	2,0128956	2,012895599

unpaired	**VGLUT1 Brightness**							
two tailed t-test	CENTII	CENT III	CUL	DEC	FOTU	PYR	UVU	NOD
p-value	0,00272728	0,20836985	0,12807517	0,363966355	0,023650355	0,126783174	0,0081174	0,000119907
Degree of Freedom	df 34	df 34	df 34	df 34	df 34	df 34	df 34	df 34
T-Value	2,03224451	2,03224451	2,03224451	2,032244509	2,032244509	2,032244509	2,0322445	2,032244509

unpaired	**VGLUT1 Brightness**			unpaired	**VGLUT1 Counts**		unpaired	**VGLUT1 Density**
two tailed t-test	all			two tailed t-test	CENTII		two tailed t-t	CENTII
p-value	4,4805E-08			p-value	1,52342E-06		p-value	0,000678631
Degree of Freedom	df 286			Degree of Freedom	df 34		Degree of Fre	df 34
T-Value	1,96829326			T-Value	2,032244509		T-Value	2,032244509

unpaired	**Branching**	**Area**	% Area		unpaired	**Dendrite width**		
two tailed t-test		CENTIII	CENTIII		two tailed t-test		CENTIII	CUL
p-value		0,92252913	0,45532465		p-value		1,692E-29	2,33828E-16
Degree of Freedom		df 33	df 33		Degree of Freedom		df 177	df 169
T-Value		2,0345153	2,0345153		T-Value		1,9734572	1,974100447

unpaired	**Parv counts**		
two tailed t-test		CENTII	UVU
p-value		0,22765282	0,58176019
Degree of Freedom		df 30	df 31
T-Value		2,04227246	2,03951345

WT vs. Gαo1 KO

Statistical Report

WT vs. Gαo2 KO

unpaired						
two tailed t-test	Cerebellum area	Length	Height	ML Area	GCL Area	
p-value	0,0006	1,41E-02	0,0059	0,5964	0,2237	
Degree of Freedom	df 62	df 60	df 60	df 62	df 62	
T-Value	1,99897152	2,00029782	2,00029782	1,99897152	1,99897152	

unpaired	Molecular layer thickness							
two tailed t-test	CENTII	CENT III	CUL	DEC	FOTU	PYR	UVU	NOD
p-value	4,6823E-15	2,0359E-10	3,2559E-05	0,05559654	0,00968695	1,7559E-07	0,00234981	3,5727E-05
Degree of Freedom	df 62	df 62	df 62	df 62	df 62	df 62	df 62	df 62
T-Value	1,99897152	1,99897152	1,99897152	1,99897152	1,99897152	1,99897152	1,99897152	1,99897152

unpaired	Molecular layer Area							
two tailed t-test	CENTII	CENT III	CUL	DEC	FOTU	PYR	UVU	NOD
p-value	2,16E-07	0,0474	7,51E-05	0,0624	0,0708	0,3586	0,0000254	0,0054
Degree of Freedom	df 62	df 62	df 62	df 62	df 62	df 62	df 62	df 62
T-Value	1,99897152	1,99897152	1,99897152	1,99897152	1,99897152	1,99897152	1,99897152	1,99897152

unpaired	Granule Cell layer thickness							
two tailed t-test	CENTII	CENT III	CUL	DEC	FOTU	PYR	UVU	NOD
p-value	0,07510053	3,5748E-07	0,00940583	0,43631039	0,01545649	0,00035852	0,07893422	0,02444525
Degree of Freedom	df 38	df 38	df 38	df 38	df 38	df 38	df 38	df 38
T-Value	2,02439416	2,02439416	2,02439416	2,02439416	2,02439416	2,02439416	2,02439416	2,02439416

unpaired	Granule Cell layer Area							
two tailed t-test	CENTII	CENT III	CUL	DEC	FOTU	PYR	UVU	NOD
p-value	8,8505E-06	0,17155386	2,9162E-05	0,02005974	0,39459281	0,08896607	6,0714E-05	0,21108957
Degree of Freedom	df 62	df 62	df 62	df 62	df 62	df 62	df 62	df 62
T-Value	1,99897152	1,99897152	1,99897152	1,99897152	1,99897152	1,99897152	1,99897152	1,99897152

		Gao2 Expression						
WT NOD agai	unpaired							
other WT folia	two tailed t-te	CENTII	CENT III	CUL	DEC	FOTU	PYR	UVU
	p-value	0,50935555	0,00819999	0,00199898	0,00034127	0,00031883	0,00574428	0,5701945
	Degree of Free	df 36	df 36	df 36	df 36	df 36	df 36	df 36
	T-Value	2,028094	2,028094	2,028094	2,028094	2,028094	2,028094	2,028094

WT vs. Gαo2 KO

unpaired			
two tailed t-test	VGLUT2 Count total	VGLUT2 Count Density	VGLUT2 Count Size
p-value	7,408E-06	0,00048958	0,06409753
Degree of Freedom	df 54	df 54	df 54
T-Value	2,00487929	2,00487929	2,00487929

unpaired	VGLUT2 counts							
two tailed t-test	CENTII	CENT III	CUL	DEC	FOTU	PYR	UVU	NOD
p-value	4,5576E-12	0,00625671	0,77430502	0,8844576	0,60898279	0,05436731	0,0003278	0,00241222
Degree of Freedom	df 54	df 54	df 54	df 54	df 54	df 54	df 54	df 54
T-Value	2,00487929	2,00487929	2,00487929	2,00487929	2,00487929	2,00487929	2,0048793	2,00487929

unpaired	VGLUT2 density							
two tailed t-test	CENTII	CENT III	CUL	DEC	FOTU	PYR	UVU	NOD
p-value	0,00444517	0,44012347	0,86787614	0,20898377	0,3458285	0,51176611	0,006496	0,10502033
Degree of Freedom	df 54	df 54	df 54	df 54	df 54	df 54	df 54	df 54
T-Value	2,00487929	2,00487929	2,00487929	2,00487929	2,00487929	2,00487929	2,0048793	2,00487929

unpaired	VGLUT2 size							
two tailed t-test	CENTII	CENT III	CUL	DEC	FOTU	PYR	UVU	NOD
p-value	0,00934992	0,35386658	0,7765402	0,27188175	0,16056333	0,0374896	0,0235914	0,88196806
Degree of Freedom	df 54	df 54	df 54	df 54	df 54	df 54	df 54	df 54
T-Value	2,00487929	2,00487929	2,00487929	2,00487929	2,00487929	2,00487929	2,0048793	2,00487929

unpaired			
two tailed t-test	VGAT Count total	VGAT Count Density	VGAT Count Size
p-value	0,00050103	0,50303964	0,28638842
Degree of Freedom	df 58	df 58	df 58
T-Value	2,00171748	2,00171748	2,00171748

unpaired	VGAT counts							
two tailed t-test	CENTII	CENT III	CUL	DEC	FOTU	PYR	UVU	NOD
p-value	0,00022647	6,2992E-07	0,15772908	0,83097394	0,49909321	0,18004195	0,0164984	0,70250837
Degree of Freedom	df 58	df 58	df 58	df 58	df 58	df 58	df 58	df 58
T-Value	2,00171748	2,00171748	2,00171748	2,00171748	2,00171748	2,00171748	2,0017175	2,00171748

unpaired	VGAT density							
two tailed t-test	CENTII	CENT III	CUL	DEC	FOTU	PYR	UVU	NOD
p-value	0,12275917	0,04936711	0,20018904	0,89087649	0,90959937	0,00175524	0,2242681	0,94545997
Degree of Freedom	df 58	df 58	df 58	df 58	df 58	df 58	df 58	df 58
T-Value	2,00171748	2,00171748	2,00171748	2,00171748	2,00171748	2,00171748	2,0017175	2,00171748

unpaired	VGAT size							
two tailed t-test	CENTII	CENT III	CUL	DEC	FOTU	PYR	UVU	NOD
p-value	0,000762	0,4403972	0,59724863	0,20350113	0,92719905	0,98378757	0,0253624	0,49225278
Degree of Freedom	df 58	df 58	df 58	df 58	df 58	df 58	df 58	df 58
T-Value	2,00171748	2,00171748	2,00171748	2,00171748	2,00171748	2,00171748	2,0017175	2,00171748

unpaired	VGLUT1 Brightness							
two tailed t-test	CENTII	CENT III	CUL	DEC	FOTU	PYR	UVU	NOD
p-value	0,0955734	0,93467011	0,76675437	0,38523442	0,58246964	0,96027429	0,0056229	0,37792306
Degree of Freedom	df 34	df 34	df 34	df 34	df 34	df 34	df 34	df 34
T-Value	2,03224451	2,03224451	2,03224451	2,03224451	2,03224451	2,03224451	2,0322445	2,03224451

unpaired	VGLUT1 Brightness		unpaired	VGLUT1 Counts		unpaired	VGLUT1 Density
two tailed t-test	all		two tailed t-te	UVU		two tailed t-t	UVU
p-value	0,18588147		p-value	0,6664548		p-value	0,74064128
Degree of Freedom	df 259		Degree of Free	df 30		Degree of Fre	df 30
T-Value	1,96916556		T-Value	2,04227246		T-Value	2,04227246

unpaired	Branching	Area	% Area		unpaired	Dendrite width		
two tailed t-test		UVU	UVU		two tailed t-test		CENTIII	UVU
p-value		0,00105791	0,00051445		p-value		0,0033771	0,02574181
Degree of Freedom		df 33	df 33		Degree of Freedom		df 152	df 141
T-Value		2,0345153	2,0345153		T-Value		1,9756939	1,97693149

unpaired	Parv counts		
two tailed t-test		CENTIII	PYR
p-value		0,46329895	0,01610395
Degree of Freedom		df 30	df 26
T-Value		2,04227246	2,05552944

WT vs. Gαo2 KO

Statistical Report

WT vs. Gαo KO

unpaired						
two tailed t-test	**Cerebellum area**	**Length**	**Height**		**ML Area**	**GCL Area**
p-value	8,82204E-07	5,61E-09	1,7877E-11		0,0191	0,2491
Degree of Freedom	df 60	df 60	df 60		df 54	df 54
T-Value	2,000297822	2,00029782	2,00029782		2,00487929	2,00487929

unpaired	**Molecular layer thickness**							
two tailed t-test	CENTII	CENT III	CUL	DEC	FOTU	PYR	UVU	NOD
p-value	3,70652E-13	3,641E-15	0,01029599	4,8571E-08	0,00448731	8,7411E-08	0,18048072	3,17374E-06
Degree of Freedom	df 62	df 62	df 62	df 62	df 62	df 62	df 62	df 62
T-Value	1,998971517	1,99897152	1,99897152	1,99897152	1,99897152	1,99897152	1,99897152	1,998971517

unpaired	**Molecular layer Area**							
two tailed t-test	CENTII	CENT III	CUL	DEC	FOTU	PYR	UVU	NOD
p-value	4,33E-02	0,2007	9,76E-07	1,49E-12	1,84E-06	1,04E-05	7,79E-02	0,0003
Degree of Freedom	df 62	df 62	df 62	df 62	df 62	df 62	df 62	df 62
T-Value	1,998971517	1,99897152	1,99897152	1,99897152	1,99897152	1,99897152	1,99897152	1,998971517

unpaired	**Granule Cell layer thickness**							
two tailed t-test	CENTII	CENT III	CUL	DEC	FOTU	PYR	UVU	NOD
p-value	0,006747599	8,0043E-08	0,00257965	0,28547839	0,00021503	0,07897017	0,43944851	0,378386603
Degree of Freedom	df 62	df 62	df 62	df 62	df 62	df 62	df 62	df 62
T-Value	1,998971517	1,99897152	1,99897152	1,99897152	1,99897152	1,99897152	1,99897152	1,998971517

unpaired	**Granule Cell layer Area**							
two tailed t-test	CENTII	CENT III	CUL	DEC	FOTU	PYR	UVU	NOD
p-value	0,5391	0,491	8,77E-06	1,21E-09	4,73E-06	6,20E-01	4,41E-01	1,67E-01
Degree of Freedom	df 62	df 62	df 62	df 62	df 62	df 62	df 62	df 62
T-Value	1,998971517	1,99897152	1,99897152	1,99897152	1,99897152	1,99897152	1,99897152	1,998971517

		**Gao Expression**						
WT NOD against	unpaired							
other WT folia	two tailed t-te	CENTII	CENT III	CUL	DEC	FOTU	PYR	UVU
	p-value	2,6608E-06	1,276E-09	1,5041E-10	7,0881E-10	9,165E-12	3,42092E-07	0,0655134
	Degree of Free	df 36	df 36	df 36	df 36	df 36	df 36	df 36
	T-Value	2,028094	2,028094	2,028094	2,028094	2,028094	2,028094001	2,028094

WT vs. Gαo KO

unpaired						
two tailed t-test	**VGLUT2 Count total**		**VGLUT2 Count Density**		**VGLUT2 Count Size**	
p-value	2,6474E-32		5,037E-16		7,8387E-08	
Degree of Freedom	df 60		df 60		df 60	
T-Value	2,00029782		2,00029782		2,00029782	

unpaired	**VGLUT2 counts**							
two tailed t-test	CENTII	CENT III	CUL	DEC	FOTU	PYR	UVU	NOD
p-value	1,9687E-06	2,549E-05	8,9342E-12	2,0983E-10	0,00577966	0,00023327	2,3197E-05	0,0198975
Degree of Freedom	df 60	df 60	df 60	df 60	df 60	df 60	df 60	df 60
T-Value	2,00029782	2,00029782	2,00029782	2,00029782	2,00029782	2,00029782	2,00029782	2,00029782

unpaired	**VGLUT2 density**							
two tailed t-test	CENTII	CENT III	CUL	DEC	FOTU	PYR	UVU	NOD
p-value	0,01388028	0,00090531	1,7713E-11	2,9865E-07	0,81275348	0,07168772	0,0002172	0,15748617
Degree of Freedom	df 60	df 60	df 60	df 60	df 60	df 60	df 60	df 60
T-Value	2,00029782	2,00029782	2,00029782	2,00029782	2,00029782	2,00029782	2,00029782	2,00029782

unpaired	**VGLUT2 size**							
two tailed t-test	CENTII	CENT III	CUL	DEC	FOTU	PYR	UVU	NOD
p-value	0,02681212	0,03173494	8,481E-15	0,00029605	0,20590188	0,11446252	0,00412668	0,05374547
Degree of Freedom	df 60	df 60	df 60	df 60	df 60	df 60	df 60	df 60
T-Value	2,00029782	2,00029782	2,00029782	2,00029782	2,00029782	2,00029782	2,00029782	2,00029782

unpaired						
two tailed t-test	**VGAT Count total**		**VGAT Count Density**		**VGAT Count Size**	
p-value	5,5611E-13		9,3654E-10		4,7927E-05	
Degree of Freedom	df 46		df 46		df 46	
T-Value	2,0128956		2,0128956		2,0128956	

unpaired								
two tailed t-test	**VGAT counts**							
p-value	CENTII	CENT III	CUL	DEC	FOTU	PYR	UVU	NOD
Degree of Freedom	0,00541565	0,01275532	0,00010548	2,7565E-05	0,00034066	0,66772066	0,00047497	0,04067283
T-Value	df 46	df 46	df 46	df 46	df 46	df 46	df 46	df 46
	2,0128956	2,0128956	2,0128956	2,0128956	2,0128956	2,0128956	2,0128956	2,0128956

unpaired	**VGAT density**							
two tailed t-test	CENTII	CENT III	CUL	DEC	FOTU	PYR	UVU	NOD
p-value	0,5280958	0,00469203	0,00020295	6,4295E-05	0,35407948	0,83253758	0,01343274	0,02300442
Degree of Freedom	df 46	df 46	df 46	df 46	df 46	df 46	df 46	df 46
T-Value	2,0128956	2,0128956	2,0128956	2,0128956	2,0128956	2,0128956	2,0128956	2,0128956

unpaired	**VGAT size**							
two tailed t-test	CENTII	CENT III	CUL	DEC	FOTU	PYR	UVU	NOD
p-value	0,71934087	0,01371555	4,6883E-06	0,01013735	0,2730457	0,6264686	0,02750647	0,42233755
Degree of Freedom	df 46	df 46	df 46	df 46	df 46	df 46	df 46	df 46
T-Value	2,0128956	2,0128956	2,0128956	2,0128956	2,0128956	2,0128956	2,0128956	2,0128956

unpaired	**VGLUT1 Brightness**							
two tailed t-test	CENTII	CENT III	CUL	DEC	FOTU	PYR	UVU	NOD
p-value	0,02965564	0,02491066	7,6561E-05	8,7092E-07	2,5128E-05	0,00399288	0,01299858	0,15506799
Degree of Freedom	df 34	df 34	df 34	df 34	df 34	df 34	df 34	df 34
T-Value	2,03224451	2,03224451	2,03224451	2,03224451	2,03224451	2,03224451	2,03224451	2,03224451

unpaired	**VGLUT1 Brightness**			unpaired	**VGLUT1 Counts**		unpaired	**VGLUT1 Density**
two tailed t-test	all			two tailed t-te	DEC		two tailed t-te	DEC
p-value	3,3406E-15			p-value	0,00086015		p-value	0,00086015
Degree of Freedom	df 276			Degree of Fre	df 30		Degree of Fre	df 30
T-Value	1,96859634			T-Value	2,04227246		T-Value	2,04227246

unpaired	**Branching**	**Area**	**% Area**		unpaired	**Dendrite width**		
two tailed t-test		DEC	DEC		two tailed t-test		CENTIII	DEC
p-value		5,8564E-05	8,2198E-05		p-value		1,0859E-29	2,4E-19
Degree of Freedom		df 34	df 34		Degree of Freedom		df 124	df 102
T-Value		2,03224451	2,03224451		T-Value		1,97928012	1,98349526

unpaired	**Parv counts**		
two tailed t-test		CENTIII	CUL
p-value		0,7772488	0,371257
Degree of Freedom		df 33	df 29
T-Value		2,0345153	2,04522964

WT vs. Gαo KO

Statistical Report
